# Identification and verification of a novel signature that combines cuproptosis-related genes with ferroptosis-related genes in osteoarthritis using bioinformatics analysis and experimental validation

**DOI:** 10.1186/s13075-024-03328-3

**Published:** 2024-05-13

**Authors:** Baoqiang He, Yehui Liao, Minghao Tian, Chao Tang, Qiang Tang, Fei Ma, Wenyang Zhou, Yebo Leng, Dejun Zhong

**Affiliations:** 1 Department of Orthopedics, The Affiliated Hospital of Southwest Medical University, No. 25 Taping Street, Lu Zhou City, China; 2 Southwest Medical University, Lu Zhou City, China; 3 Meishan Tianfu New Area People’s Hospital, Meishan City, China

**Keywords:** Osteoarthritis, Cuproptosis, Ferroptosis, Machine learning, Bioinformatics

## Abstract

**Background:**

Exploring the pathogenesis of osteoarthritis (OA) is important for its prevention, diagnosis, and treatment. Therefore, we aimed to construct novel signature genes (c-FRGs) combining cuproptosis-related genes (CRGs) with ferroptosis-related genes (FRGs) to explore the pathogenesis of OA and aid in its treatment.

**Materials and methods:**

Differentially expressed c-FRGs (c-FDEGs) were obtained using R software. Enrichment analysis was performed and a protein–protein interaction (PPI) network was constructed based on these c-FDEGs. Then, seven hub genes were screened. Three machine learning methods and verification experiments were used to identify four signature biomarkers from c-FDEGs, after which gene set enrichment analysis, gene set variation analysis, single-sample gene set enrichment analysis, immune function analysis, drug prediction, and ceRNA network analysis were performed based on these signature biomarkers. Subsequently, a disease model of OA was constructed using these biomarkers and validated on the GSE82107 dataset. Finally, we analyzed the distribution of the expression of these c-FDEGs in various cell populations.

**Results:**

A total of 63 FRGs were found to be closely associated with 11 CRGs, and 40 c-FDEGs were identified. Bioenrichment analysis showed that they were mainly associated with inflammation, external cellular stimulation, and autophagy. CDKN1A, FZD7, GABARAPL2, and SLC39A14 were identified as OA signature biomarkers, and their corresponding miRNAs and lncRNAs were predicted. Finally, scRNA-seq data analysis showed that the differentially expressed c-FRGs had significantly different expression distributions across the cell populations.

**Conclusion:**

Four genes, namely CDKN1A, FZD7, GABARAPL2, and SLC39A14, are excellent biomarkers and prospective therapeutic targets for OA.

**Supplementary Information:**

The online version contains supplementary material available at 10.1186/s13075-024-03328-3.

## Introduction

As a degenerative disease that is difficult to reverse, osteoarthritis (OA) is often accompanied by joint pain, stiffness, joint swelling, restricted movement, and joint deformity, all of which seriously affect daily life activities. The structural changes in OA mainly involve the articular cartilage, subchondral bone, ligaments, capsule, synovium, and periarticular muscles [1]. The prevalence of OA is steadily rising due to the aging population and the obesity epidemic [1], and it has placed a significant burden on society [2]. Currently, the main treatments for OA remain nonsteroidal anti-inflammatory drugs (NSAIDs), pain medications, and joint replacement surgery. However, these treatments cannot reduce the incidence of the early stages of the disease [3], prevent further cartilage degeneration, or promote cartilage regeneration [4]. Therefore, further understanding of the pathophysiological mechanisms of OA could aid in the development of additional approaches for more effective diagnosis and treatment.

Ferroptosis is a specific type of programmed cell death driven by iron-dependent lipid peroxidation characterized by an abnormal accumulation of lipid reactive oxygen species (ROS) [5, 6]. This programmed cell death was first reported and named by Dixon in 2012 [7]. Many studies have demonstrated that ferroptosis and the development of OA are closely related [8–11], and ferroptosis-related genes (FRGs) can help in the diagnosis of OA, as well as in predicting the immune status of patients with OA [12, 13].

Copper is an indispensable trace element involved in a wide range of biological reactions. A small study reported elevated plasma and synovial copper concentrations in patients with OA compared with healthy controls [14], and another study also found that elevated levels of copper were associated with an increased risk of OA [15]. When the oxidizing capacity of copper ions in the body exceeds the antioxidant capacity of the body, joints can be destroyed [16]. Cuproptosis is a novel form of programmed cell death during which copper binds directly to the fatty acylated components of the tricarboxylic acid (TCA) cycle, thereby leading to an increase in toxic proteins and ultimately to cell death [17]. Ferroptosis is an iron-dependent programmed cell death caused by lipid peroxidation and the massive accumulation of reactive oxygen radicals[7]. Furthermore, copper and iron are closely related; copper is essential for iron absorption, meaning that copper deficiency or overload can impair the balance of iron metabolism [18]. When the balance of iron metabolism is disturbed, lipid peroxidation and oxidative stress may be induced, which in turn leads to ferroptosis and alters the expression of FRGs [19–21]. However, it has not yet been reported whether new signature genes (c-FRGs) combining cuproptosis-related genes (CRGs) with FRGs are beneficial for the diagnosis and treatment of OA.

In this study, we explored and analyzed the immune characteristics and biological functions of c-FRGs in patients with OA. In addition, we screened key ferroptosis-related biomarkers associated with cuproptosis in OA, constructed ceRNA networks, and predicted potential drugs for OA treatment. Our results suggest that c-FRGs may play an important role in the pathophysiological process of OA and provide new directions and ideas for OA research.

## Materials and methods

### Data collection

The US National Center for Biotechnology Information (NCBI) gene expression omnibus (GEO) is the world's largest international public repository of high-throughput molecular information. Using “osteoarthritis” as a search term, the GEO database (https://www.ncbi.nlm.nih.gov/geo/) was searched for appropriate datasets, and four datasets that met the study requirements were downloaded. These four datasets were GSE55235, GSE169077, GSE55457, and GSE55584, and the chip type was Affymetrix Human Genome U133a. We eventually obtained 25 normal human synovial samples and 32 OA synovial samples from the four datasets as samples for the follow-up study. To assess the accuracy of the analysis, the GSE82107 dataset was used as validation sets. In addition, the FRGs and CRGs were obtained from the published literature [6] and the FerrDb website (http://www.zhounan.org/ferrdb/).

### Extraction of c-FRGs and obtaining differentially expressed c-FRGs

Inter-batch differences between the four groups (GSE55235, GSE169077, GSE55457, and GSE55584) were eliminated using “affy” packet merging and the “sva” packet. We performed a Pearson correlation analysis of CRGs with FRGs to obtain particular FRGs (c-FRGs) that were highly correlated with CRGs (|r| > 0.5, adj. *p* value < 0.05). Differentially expressed genes (DEGs) and differentially expressed c-FRGs (c-FDEGs) were obtained using the “limma” package (*p* value < 0.05).

### Function enrichment analysis and protein–protein interaction (PPI) networks

To acquire disease-related biological functions and signaling pathways, Gene Ontology (GO) enrichment analysis and Kyoto Encyclopedia of Genes and Genomes (KEGG) pathway enrichment analysis of c-FDEGs were performed. GO enrichment analysis was used to describe the molecular functions (MF), cellular components (CC), and biological processes (BP) involved in the target genes (*p*-value < 0.05). KEGG analysis was used to systematically analyze gene functions and to link genomic information and functional information (*p*-value < 0.05). The results of the gene set enrichment analysis (GSEA), GO enrichment analysis, and KEGG pathway enrichment analysis of the c-FDEGs were visualized using the “ClusterProfiler” package in R. GSEA was based on the gene set (h. all. v7. 5. 1. symbols. gmt), which was downloaded from MSigDB (https://www.gsea-msigdb.org/gsea/msigdb/index.jsp). The STRING database is used for searching interactions between known proteins and for predicting interactions between proteins and is one of the most data-rich and widely used databases for studying protein interactions. Protein interaction analysis was performed on all c-FDEGs through the STRING website (https://string-db.org/) and visualized using Cytoscape software. The degree values of the c-FDEGs were calculated using the cytoHubba plugin, and the top seven genes were used as hub genes.

### Acquisition and validation of biomarkers

In this research, we used three machine learning algorithms: support vector machine recursive feature elimination (SVM-RFE), least absolute shrinkage and selection operator (LASSO) regression analysis, and random forest analysis (RF). First, we used the “e1071” R package for SVM-RFE analysis. Subsequently, the “glmnet” package was used to perform LASSO regression analysis. In addition, RF was conducted adopting the “randomForest” package, and genes with importance > 1 were retained. The crossover genes obtained by these three methods were regarded as prospective biomarkers for OA.

## Construction and validation of disease model (nomogram)

In addition, a nomogram based on characteristic biomarkers was structured using the “rms” R package. Receiver operating characteristic (ROC) analysis was performed on the biomarkers and the obtained models, and the area under the curve (AUC) values were calculated with the “pROC” package to assess the diagnostic efficacy of the potential biomarkers. In addition, the four biomarkers and the obtained disease nomogram were validated on the GSE82107 validation set.

### Collection of clinical samples

Synovial tissue collection and all experimental procedures were approved by the Institutional Review Board of the Affiliated Hospital of Southwest Medical University (KY2023293) in accordance with the guidelines of the Chinese Health Sciences Administration, and written informed consent was obtained from the donors. Synovial tissue from the suprapatellar bursa was collected as OA synovial samples and normal control samples, respectively, from patients who met the American College of Rheumatology criteria for the diagnosis of primary symptomatic knee OA (n=6; men: 3, women: 3; age: 55-70 years) and from patients who underwent trauma-related lower extremity amputation but did not have osteoarthritis or rheumatoid arthritis (n=6; men: 4, women: 2; age: 50-67 years). All samples were collected within two hours of arthroplasty or lower limb amputation and were divided into two portions for subsequent immunofluorescence staining and western blot experiments, respectively.

### Immunofluorescence staining

Mid-sagittal sections (4-μm thick) of paraffin-embedded clinical synovial specimens were incubated for 1 hour at room temperature, after which the slides were closed with 10% bovine serum (Solarbio, Beijing, China) for 1 hour at room temperature and then incubated with primary antibodies for 16 hours at 4°C. The fluorescent dye was incubated for 1 hour at room temperature, and the slides were subsequently sealed with DAPI Sealer (Thermo Fisher Scientific, Waltham, MA, USA).

### Western blot analysis

Protein lysates were extracted from synovial tissue samples and lysed with RIPA buffer to extract the total protein. After conducting a BCA protein assay (Beyotime, Shanghai, China), 5 × sample buffer (Servicebio, Wuhan, China) was added to the protein lysates. Equal amounts of lysates were then separated through SDS-PAGE and transferred to a 0.22-um PVDF microporous membrane (Merck Millipore, Burlington, MA, USA). Next, the membrane was sealed with 5% skimmed milk and incubated with the primary antibody for 16 hours at 4°C, after which the membrane was incubated with the secondary antibody for 60 minutes at room temperature. Target protein bands were visualized using FDbio-Dura ECL (Merck Millipore, Burlington, MA, USA). The antibodies used for immunofluorescence and western blot in this study were as follows: rabbit anti-FZD7 (Cat. #: DF8657, 1:1,000; AFFBIOTECH, USA), rabbit anti-SLC39A14 (ZIP14) (Cat. #: 26540-1-AP, 1:1,000, Proteintech, Rosemont, IL, USA), rabbit anti-CDKN1A (p21) (Cat. #: 2947T, 1:1,000, Cell Signaling Technology, Danvers, MA, USA), rabbit anti-GABARAPL2 (Cat. #: 14256T, 1:1,000, Cell Signaling Technology), anti-GAPDH (Cat. #: 60004 -1-Ig, 1:1,000, Proteintech, USA), and species-matched HRP-conjugated secondary antibody (Cat. #: SA00001-1, 1:1,000; Proteintech, USA).

### ssGSEA, GSEA, and GSVA for differentially expressed c-FRGs

The gene set (h.all.v2022.1.Hs.symbols.gmt), a collection of 50 symbolic gene sets for humans, was downloaded from MSigDB (https://www.gsea-msigdb.org/gsea/msigdb/index.jsp). The 50 symbolic human gene set scores were calculated for each sample using single-sample GSEA (ssGSEA), and differential scores were obtained for the non-OA and OA groups. The “corrplot” package was used to perform correlation analysis between biomarkers and ssGSEA gene sets. Next, GSEA and gene set variation analysis (GSVA) were performed for the four biomarkers, the seven hub genes, and the remaining 29 differentially expressed c-FRGs.

### Prediction of therapeutic drugs

The gene–drug interaction database (DGIDB, http://www.dgidb.org) [22] can help researchers annotate known pharmacogenetic interactions and potential drug accessibility–related genes. In this research, we used DGIdb to filter potential drugs targeted to biomarkers so as to identify new therapeutic targets. The obtained drug prediction results were also imported into Cytoscape (v3.9.1) software for visualization.

### Construction of ceRNA network

The miRanda, TargetScan, and miRDB databases are authoritative databases used for predicting miRNA–target gene regulatory relationships, and spongeScan is a web tool designed for sequence-based complementary detection of miRNA-binding elements in lncRNA sequences. Biomarkers of common mRNA–miRNA interactions were identified in miRanda (http://www.microrna.org/microrna/home.do), TargetScan (http://www.targetscan.org), and miRDB (https://mirdb.org). miRNA–lncRNA interactions were obtained from Spongescan (http://spongescan.rc.ufl.edu). These interactions were imported into Cytoscape to construct the ceRNA network.

### Immune infiltration analysis

To better understand the changes that occur in the immune system of patients with OA, the “CIBERSORT” R package was used to describe the basic expression of 22 immune cell subtypes. Next, we analyzed the correlation between potential biomarkers, hub genes, and the 22 immune cell types.

### scRNA‑seq analysis

The OA synovial scRNA-seq data (GSE152805) from three patients were obtained from the GEO database and analyzed using the "Seurat" software package. To ensure high quality of the data, we removed low-quality cells (cells with <200 or >10,000 detected genes, >10% of mitochondrial genes, or <300 or >30,000 expressed genes) and low-expressed genes (any gene expressed in less than three cells). We used the "NormalizeData" function to normalize the gene expression of the included cells and performed principal component analysis (PCA) using the top 2000 highly variable genes to extract the top 12 principal components (PCs), which were retained for further analysis using the "FindVariableFeatures" function. To perform unsupervised and unbiased clustering of cell subpopulations, the "FindNeighbors," "FindClusters" (resolution = 0.6), and "RunUMAP" functions were applied. Each cell cluster was manually annotated according to the cell-specific marker genes. These marker genes were obtained from previously published literature[23, 24] and from the CellMarker website (http://xteam.xbio.top/CellMarker/). Finally, we used CellChat (1.6.1) for the inference and analysis of cell–cell communication.

## Results

**Fig. 1:**
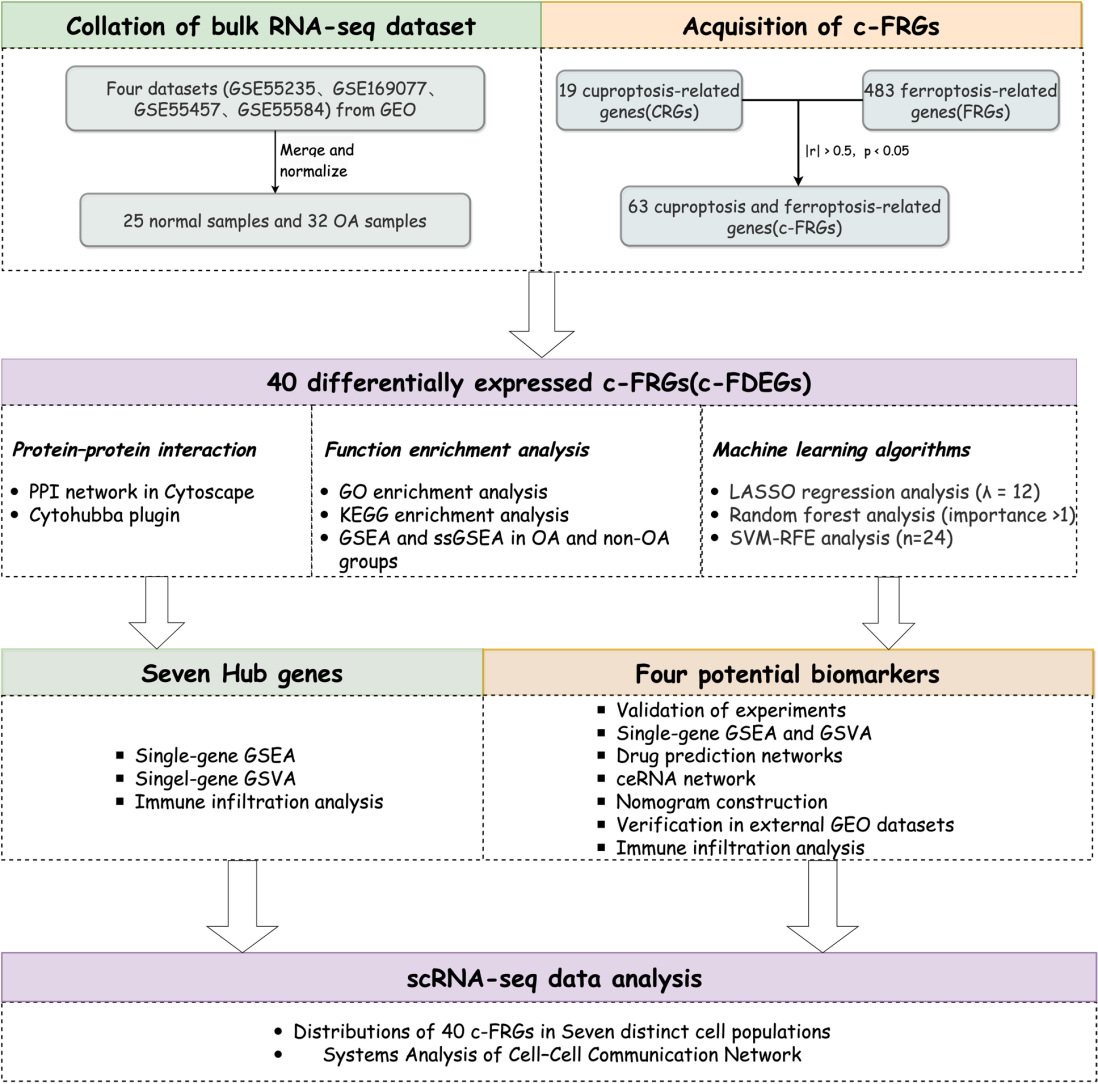
A graphical flowchart of the study design

Figure 1 describes the entire flow of the study.

### Extracting c-FRGs and obtaining differentially expressed c-FRGs

**Fig. 2:**
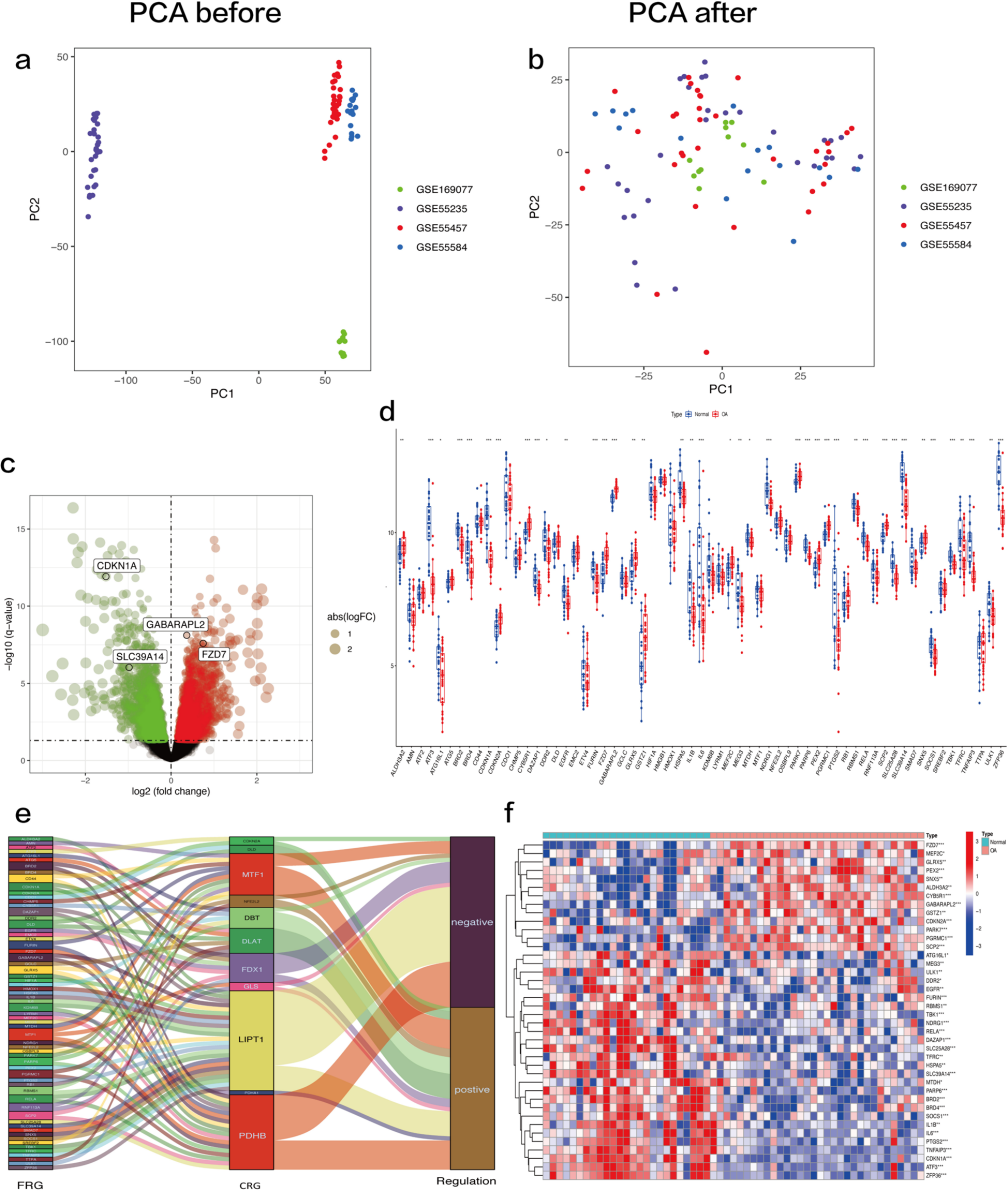
Extraction of particular ferroptosis-related genes (c-FRGs) and obtainment of differentially expressed c-FRGs (c-FDEGs). **a, b** Two-dimensional PCA cluster plot of GSE55235, GSE169077, GSE55457, and GSE55584 datasets before and after normalization. **c** Volcano plot of DEGs. Red spots represent upregulated genes and green spots represent downregulated genes. **d** Overall expression landscape of c-FRGs in osteoarthritis (OA). *P < 0.05; **P < 0.01; ***P < 0. 001. OA represents the OA group and Normal represents the normal control group. **e** Extraction of c-FDEGs. **f **Heatmap of c-FDEGs. The redder the color, the higher the expression; conversely, the bluer the color, the lower the expression

After merging the GSE55235, GSE169077, GSE55457, and GSE55584 datasets (Table 1), the newly produced gene expression matrices were subjected to normalization and presented as bidimensional PCA plots prior to and after processing (Fig. 2a and b), indicating that the final sample data obtained were plausible. A total of 63 FRGs were found to be closely associated with 11 CRGs (Fig. 2e, Supplementary Table 1). A total of 4167 DEGs were determined and identified (Fig. 2c). There were a total of 40 c-FDEGs, including 13 upregulated genes and 27 downregulated genes (Fig. 2d, Supplementary Table 2). The correlations between the 40 c-FDEGs are shown in Supplementary Figure 1. The expression patterns of the 40 c-FDEGs are visualized in the heatmap (Fig. 2f).

**Table 1:** Information of selected microarray datasets

**Accession numbers**	**Samples**			**Age (mean ± SD)**		**Sex, n (male/female)**		**Source tissue**	**Attribute**
	Platform	Normal	OA	Normal	OA	Normal	OA		
GSE55235	GPL96	10	10	N/A	N/A	N/A	N/A	Synovium	Test set (bulk RNA-seq)
GSE55457	GPL96	10	10	51±18.7	72.4±5.6	8/2	2/8	Synovium	Test set (bulk RNA-seq)
GSE55584	GPL96	0	6	N/A	73.2±7.9	N/A	0/6	Synovium	Test set (bulk RNA-seq)
GSE169077	GPL96	5	6	N/A	N/A	N/A	N/A	Synovium	Test set (bulk RNA-seq)
GSE82107	GPL570	7	10	N/A	N/A	N/A	N/A	Synovium	Validation set (bulk RNA-seq)
GSE152805	GPL20301	0	3	N/A	67.7±2.3	N/A	1/2	Synovium	Test set (scRNA-seq)

GPL96: [HG-­U133A] Affymetrix Human Genome U133A Array; GPL570: Affymetrix GeneChip Human Genome U133 Plus 2.0 Array; GPL20301: Illumina HiSeq 4000 (Homo sapiens)

*N/A* Not available, *OA* Osteoarthritis, *Bulk RNA-seq* Bulk RNA sequencing, *scRNA-seq* Single-cell RNA sequencing

### Function enrichment analysis

**Fig. 3:**
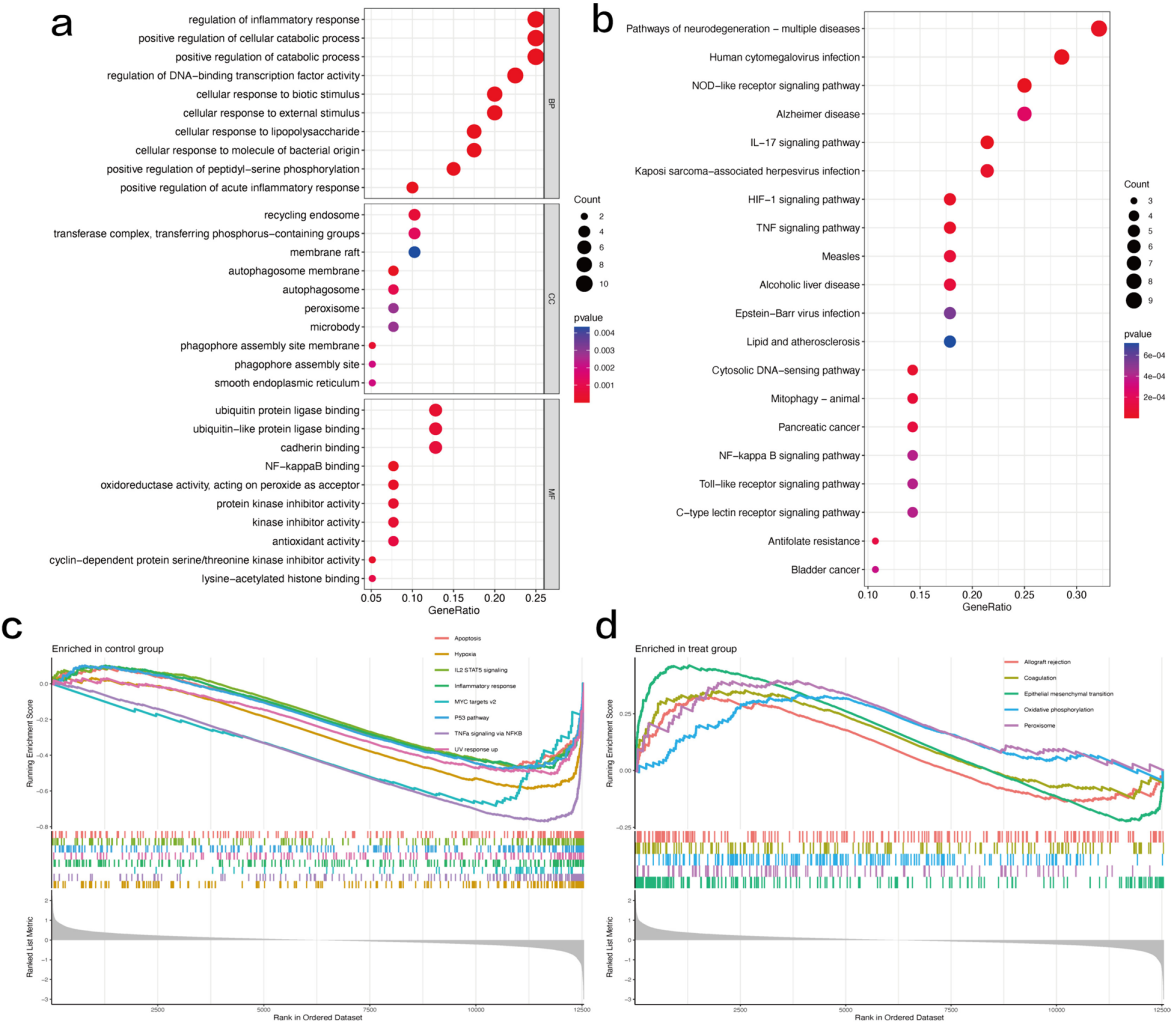
Functional analyses: (**a**) Gene Ontology (GO) enrichment analysis showed that the 40 c-FDEGs were significantly enriched in the regulation of the inflammatory response, the positive regulation of cellular catabolic process, the autophagosome membrane, the recycling endosome, and NF-κB binding. **b** Kyoto Encyclopedia of Genes and Genomes (KEGG) pathway analysis showed that these c-FDEGs were mainly involved in the IL-17 signaling pathway, NOD-like receptor signaling pathway, HIF-1 signaling pathway, and TNF signaling pathway. **c** Gene set enrichment analysis (GSEA) in the normal control group and (d) GSEA in the OA group based on the core set of 50 human genes suggested that the development of OA may be associated with hypoxia, MYC targets v2, the P53 pathway, the inflammatory response, TNFα signaling via NF-κB, the interferon-α response, and peroxisome

Understanding the signaling pathways, biological processes, and interrelationships involved in c-FDEGs is of great importance in revealing the pathogenesis of OA. GO enrichment analysis showed that c-FDEGs were significantly enriched in the regulation of the inflammatory response (BP), the positive regulation of cellular catabolic process (BP), the autophagosome membrane (CC), the recycling endosome (CC), and NF-κB binding (MF) (Fig. 3a, Supplementary Table 3). KEGG pathway analysis showed that these c-FDEGs were mainly involved in the IL-17 signaling pathway, NOD-like receptor signaling pathway, HIF-1 signaling pathway, and TNF signaling pathway (Fig. 3b, Supplementary Table 4). GSEA suggested that the development of OA may be associated with hypoxia, MYC targets v2, the P53 pathway, the inflammatory response, TNFα signaling via NF-κB, the interferon-α response, and peroxisome (Fig. 3c and d).

### Building PPI networks

**Fig. 4:**
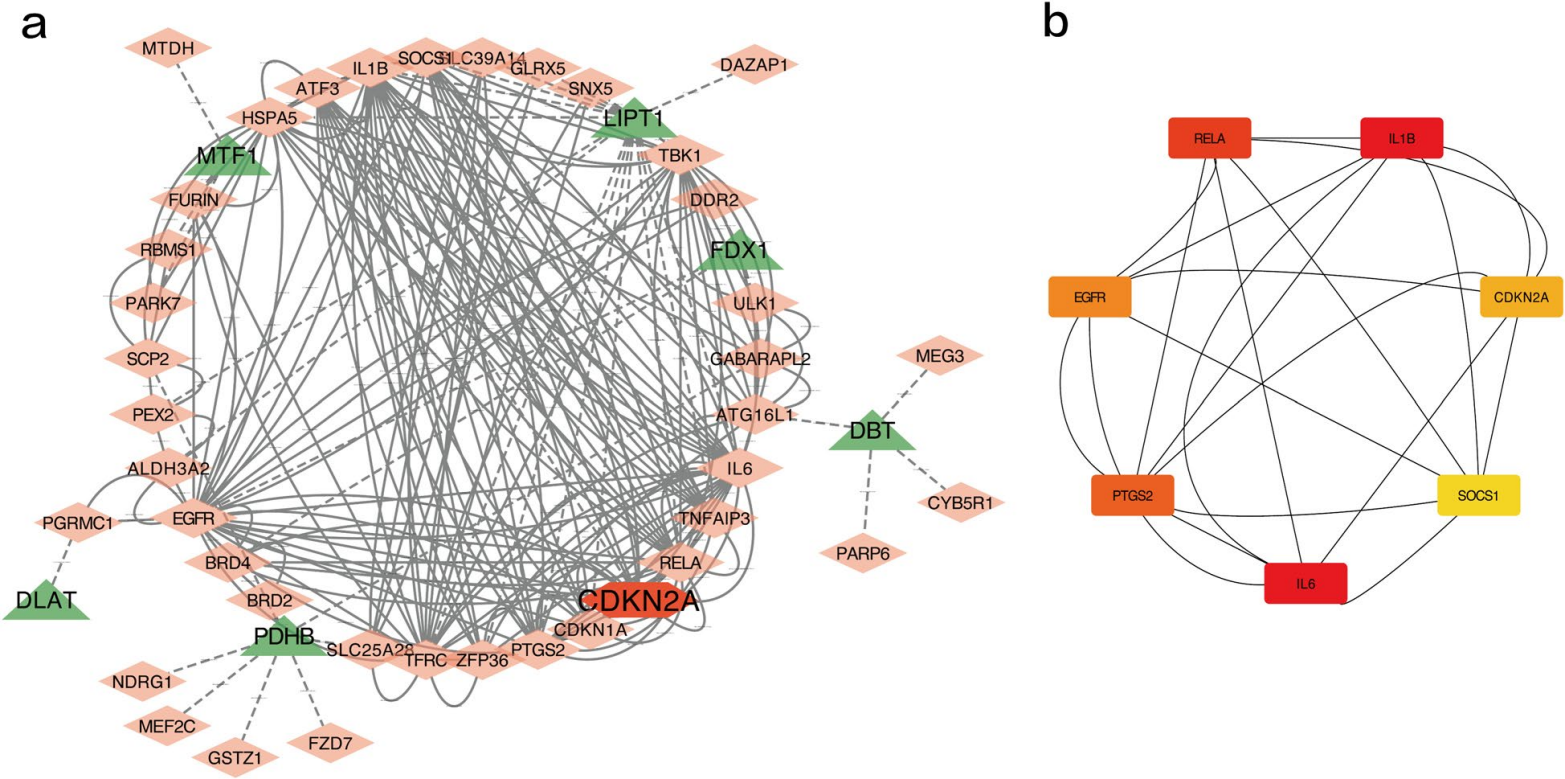
Protein–protein interaction (PPI) network and core gene screening. **a** PPI network constructed from 40 c-FDEGs; red triangles represent c-FDEGs, green triangles represent CRGs that are closely related to them, and the correlation between c-FDEGs and CRGs is indicated by dashed lines. **b** The top seven core gene interaction networks calculated using the cytoHubba plugin: the darker the color, the more powerful the critical degree

The String database is a database that can be used to retrieve interactions between known and predicted proteins. To explore the interactions between each c-FDEG, all of the abovementioned 40 c-FDEGs were imported into the STRING database. The PPI network of c-FDEGs after deleting isolated c-FDEGs and adding the six related CRGs (without CDKN2A) is shown in Fig. 4a. The cytoHubba plugin in Cytoscape software was used to calculate the degree values (degrees) of the top seven genes (IL6, IL1B, RELA, PTGS2, EGFR, CDKN2A, and SOCS1) as the PPI network’s hub genes (Fig. 4b).

### Machine learning algorithm–based biomarker screening for patients with OA

**Fig. 5:**
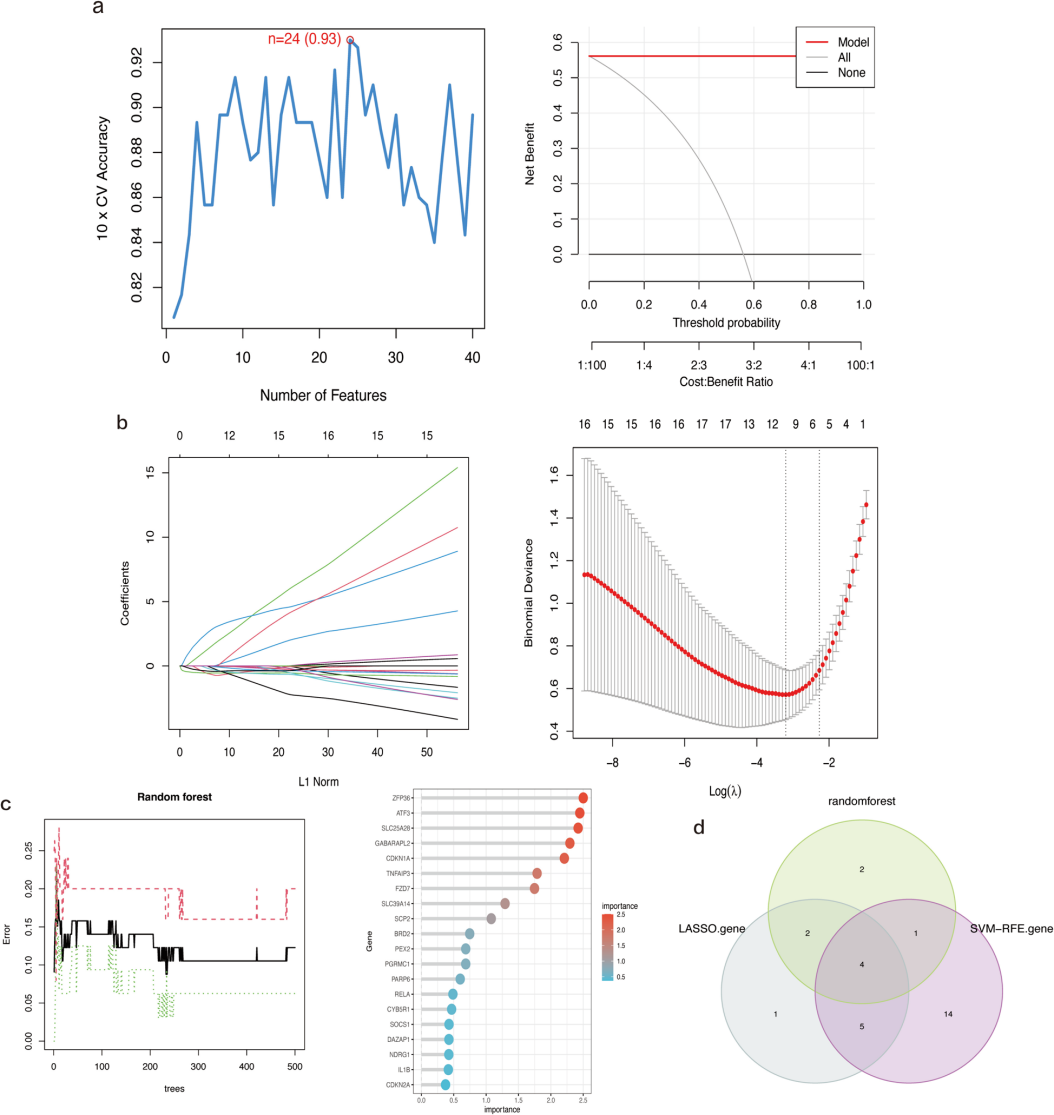
Machine learning-based potential biomarker screening. **a** SVM-RFE model with the optimal error rate when the number of signature genes was 58. **b** LASSO regression model. **c** Random forest model and the top 20 genes in terms of importance. **d** The final biomarkers screened using three machine learning algorithms

In this study, 40 c-FDEGs were further analyzed for potential biomarkers associated with OA using multiple machine learning methods. SVM-RFE analysis showed that the model containing 24 genes had the best accuracy (Fig. 5a). LASSO regression analysis showed that the model was able to accurately predict OA when λ was equal to 12. Thus, the LASSO regression model generated 12 candidate genes (Fig. 5b). We retained the candidate biomarkers with RF results importance > 1 (Fig. 5c). Lastly, the results of these three methods were integrated, and CDKN1A, FZD7, GABARAPL2, and SLC39A14 were identified as the final potential biomarkers for OA (Fig. 5d).

### Experimental validation of four biomarkers

**Fig. 6:**
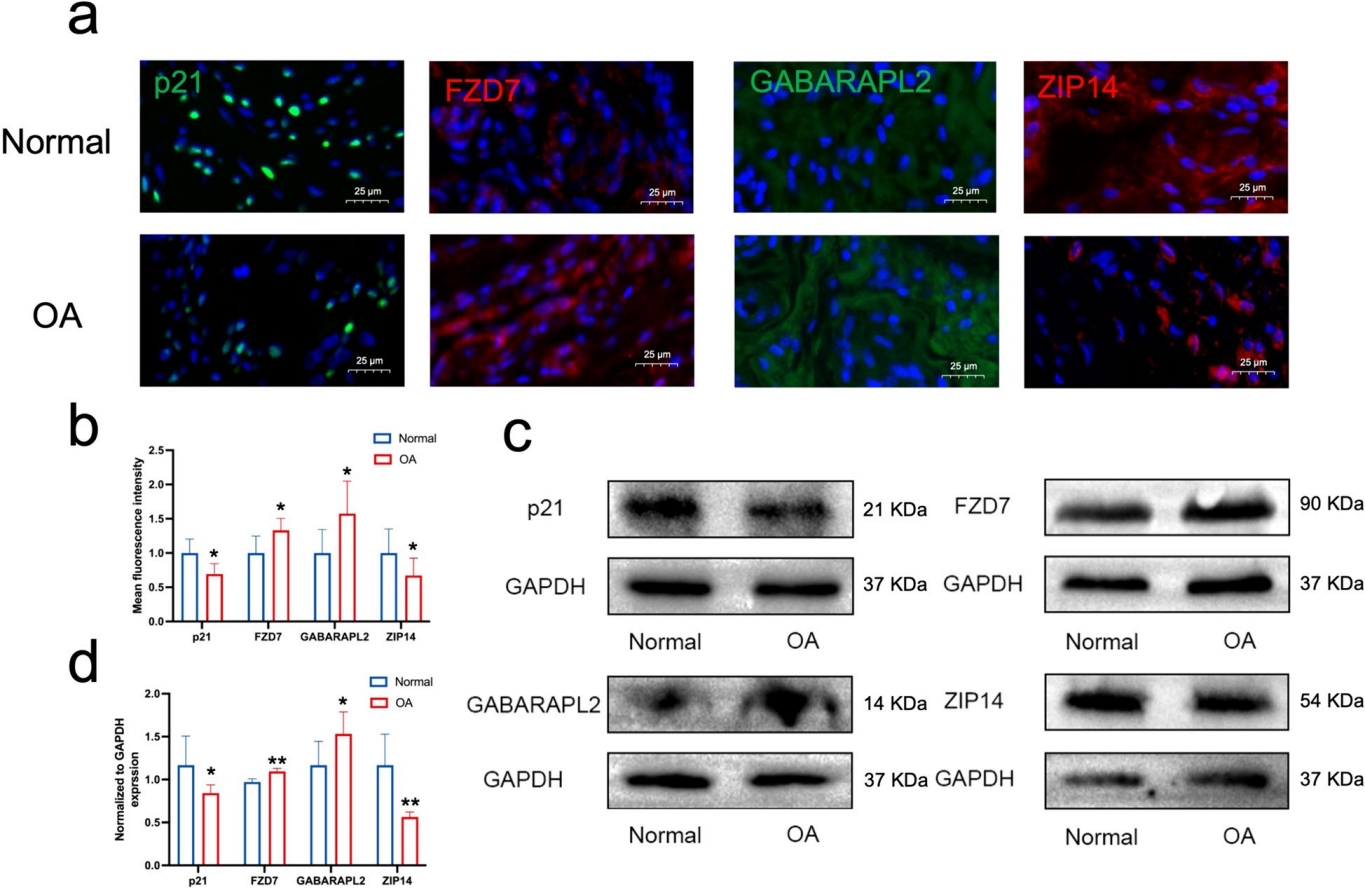
Experimental validation of four biomarkers. **a** Representative immunofluorescence staining images of the four biomarker proteins (p21, FZD7, GABARAPL2, and ZIP14) in the normal and OA groups, with nuclei stained blue with 4’,6-diamidino-2-phenylindole. Scale bar = 25 µm. **b** Semi-quantitative analysis of mean fluorescence intensity of the four biomarker proteins in the normal and OA groups (*n* = 6). (c, d) Representative western blotting and statistical comparisons of the four biomarker proteins in the normal and OA groups (*n* = 6). *p < 0.05, **p < 0.01, all by independent samples t-test

To validate the results of the bioinformatics analysis, we collected OA samples (n=6) and normal group samples (n=6), respectively, and performed western blot analysis and immunofluorescence staining (Fig. 6). Both results were consistent with the bioinformatics analysis, i.e., higher expression of FZD7 and GABARAPL2 and lower expression of CDKN1A (p21) and SLC39A14 (ZIP14) in the OA group compared with the normal group.

### ssGSEA, GSEA, and GSVA for differentially expressed c-FRGs

**Fig. 7:**
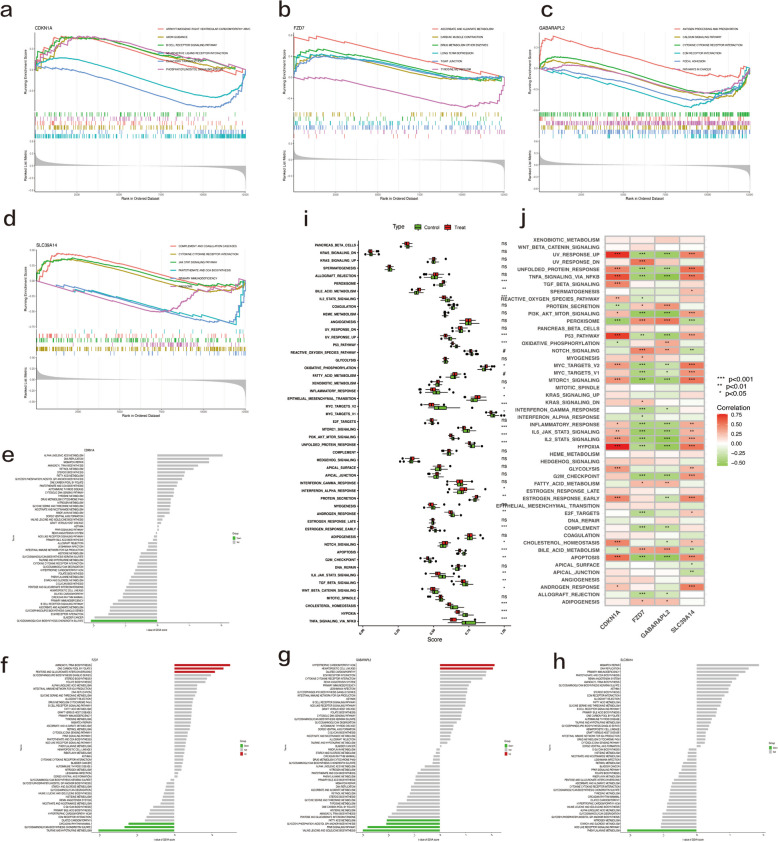
GSEA, GSVA, and ssGSEA results of four potential biomarkers. **a–d** Single-gene GSEA-KEGG pathway analysis of four potential biomarkers. We show the top six pathways with the smallest *p*-value. **e–h** High- and low-expression groups based on the expression levels of each potential biomarker combined with gene set variation analysis (GSVA). Red means the pathway is significantly upregulated, green means the pathway is significantly downregulated, and gray means the pathway is not statistically significant. **i** ssGSEA of OA and normal controls based on the h.all.v7.5.1.symbols.gmt gene set. *P < 0.05; **P < 0.01; ***P < 0. 001. Treat represents the OA group, and control represents the normal group. (j) Correlation of four biomarkers with 50 human symbolic gene sets from the h.all.v7.5.1.symbols.gmt gene set

To better capture the function of the four biomarkers in OA, GSEA, GSVA, and ssGSEA were conducted on each of the above biomarkers (Fig. 7). The ssGSEA showed that the OA group was significantly enriched in Notch signaling, interferon alpha (IFN-α) response, the Wnt/β-catenin pathway, bile acid metabolism, and peroxisome, while the non-OA group was mainly enriched in TNFα signaling via NF-κB, hypoxia, MYC targets v2, the P53 pathway, the inflammatory response, PI3K AKT mTOR signaling, and IL6 JAK STAT3 signaling (Fig. 7i). Correlation analysis showed that CDKN1A and SLC39A14 were significantly positively correlated with the gene sets of hypoxia, TNF-α signaling via NF-κB, the P53 pathway, and mTORC1 signaling. Meanwhile, GABARAPL2 and FZD7 showed significant negative correlations with the gene sets of TNF-α signaling via NF-κB, PI3K AKT mTOR signaling, and mTORC1 signaling (Fig. 7j). The single-gene GSEA results for the seven hub genes are shown in Supplementary Figure 2 (a–g). The remaining 29 differentially expressed c-FRGs are shown in Supplementary Figure 3.

### Construction and validation of disease model (nomogram)

**Fig. 8:**
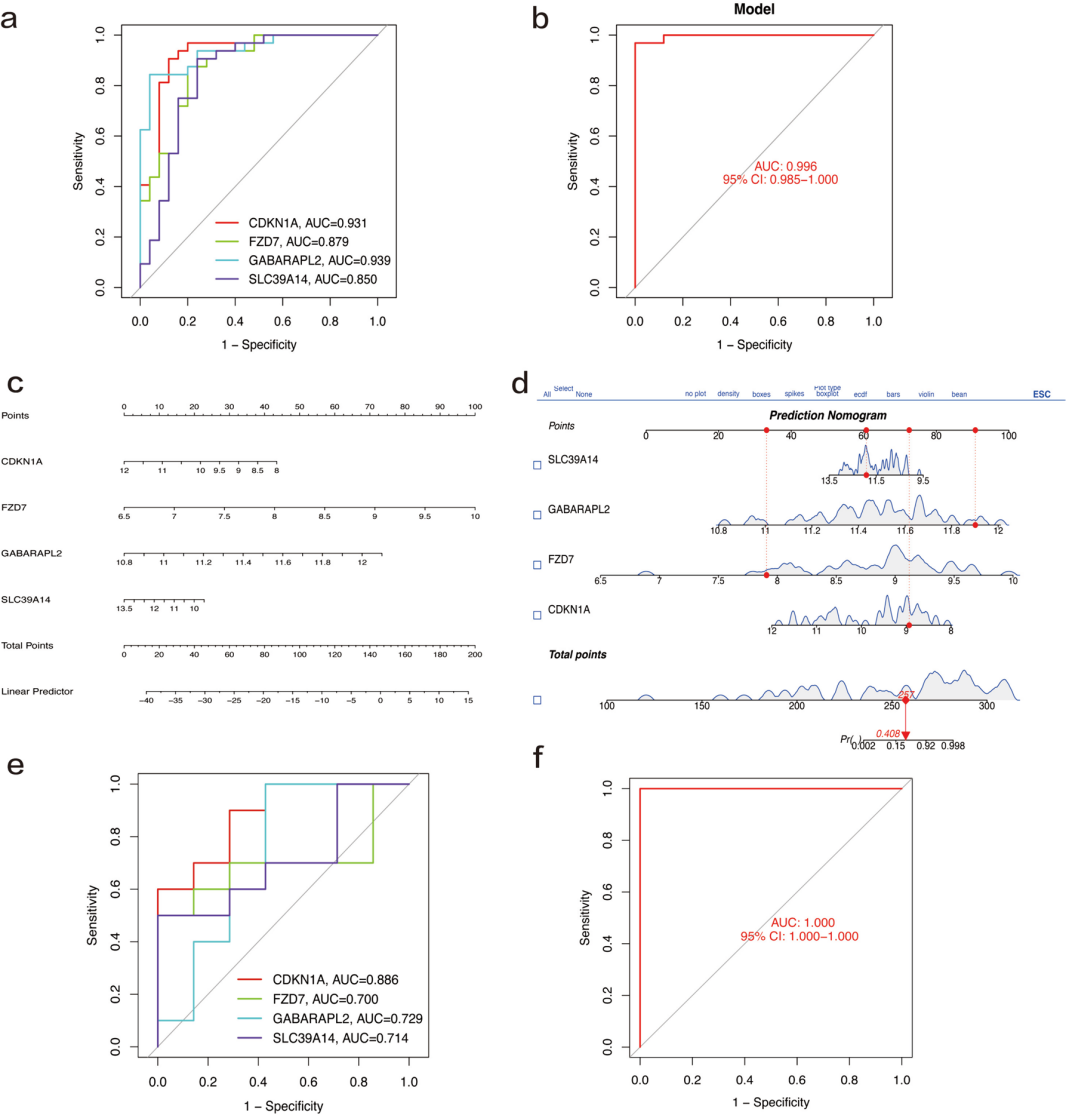
Validation of four biomarkers. **a** ROC analysis of the four biomarkers. **b** ROC analysis of the disease model constructed from the four biomarkers. **c, d** Nomograms based on the disease model: we obtained the corresponding scores for each genetic variable, drew a vertical line above the “points” axis, summed the scores of all predictor variables, found the final value on the “total score” axis, and then drew a straight line on the “probability” axis to determine the patient’s risk of osteoarthritis. **e, f** Validation of the disease model and four biomarkers on the GSE82107 validation dataset

Using the above four biomarkers, a disease nomogram was constructed. The AUC values of the individual genes CDKN1A, FZD7, GABARAPL2, and SLC39A4 were 0.931, 0.879, 0.989, and 0.850, respectively, all of which were greater than 0.85 (Fig. 8a), further indicating that the above genes had good diagnostic ability (Fig. 8b). The AUC value of this model was 0. 996, which was significantly greater than the AUC value of individual biomarkers, indicating that this model had good diagnostic value (Fig. 8c and d). To verify whether the above model is diagnostically meaningful, validation was performed on the GSE8207 dataset. The results showed that the AUC values of the four biomarkers were all greater than 0.7, and the AUC value of the model was 1 for the validation set (Fig. 8f). These results indicate that CDKN1A, FZD7, GABARAPL2, and SLC39A4 are effective disease biomarkers for OA and that the model has high diagnostic efficacy.

### Construction of drug prediction network and lncRNA–miRNA–mRNA network

The corresponding drug prediction network was constructed using the database based on the four biomarkers (Supplementary Figure 4a). The predicted drugs were celecoxib, paclitaxel, carboplatin, acetaminophen, vantictumab, and nortriptyline. Based on the competitive endogenous RNA hypothesis, an lncRNA–miRNA–mRNA competitive endogenous RNA (ceRNA) network was constructed to explore the function of lncRNA as an miRNA sponge in OA. We obtained 150 target miRNAs based on these biomarkers. Then, 48 lncRNAs were obtained based on these miRNA predictions. The four biomarkers with predicted miRNAs and lncRNAs were introduced into Cytoscape, and constituted a ceRNA network containing 48 lncRNA nodes, 150 miRNA nodes, 4 hub gene nodes, and 198 edges (Supplementary Figure 4b).

### Immune infiltration analysis

**Fig. 9:**
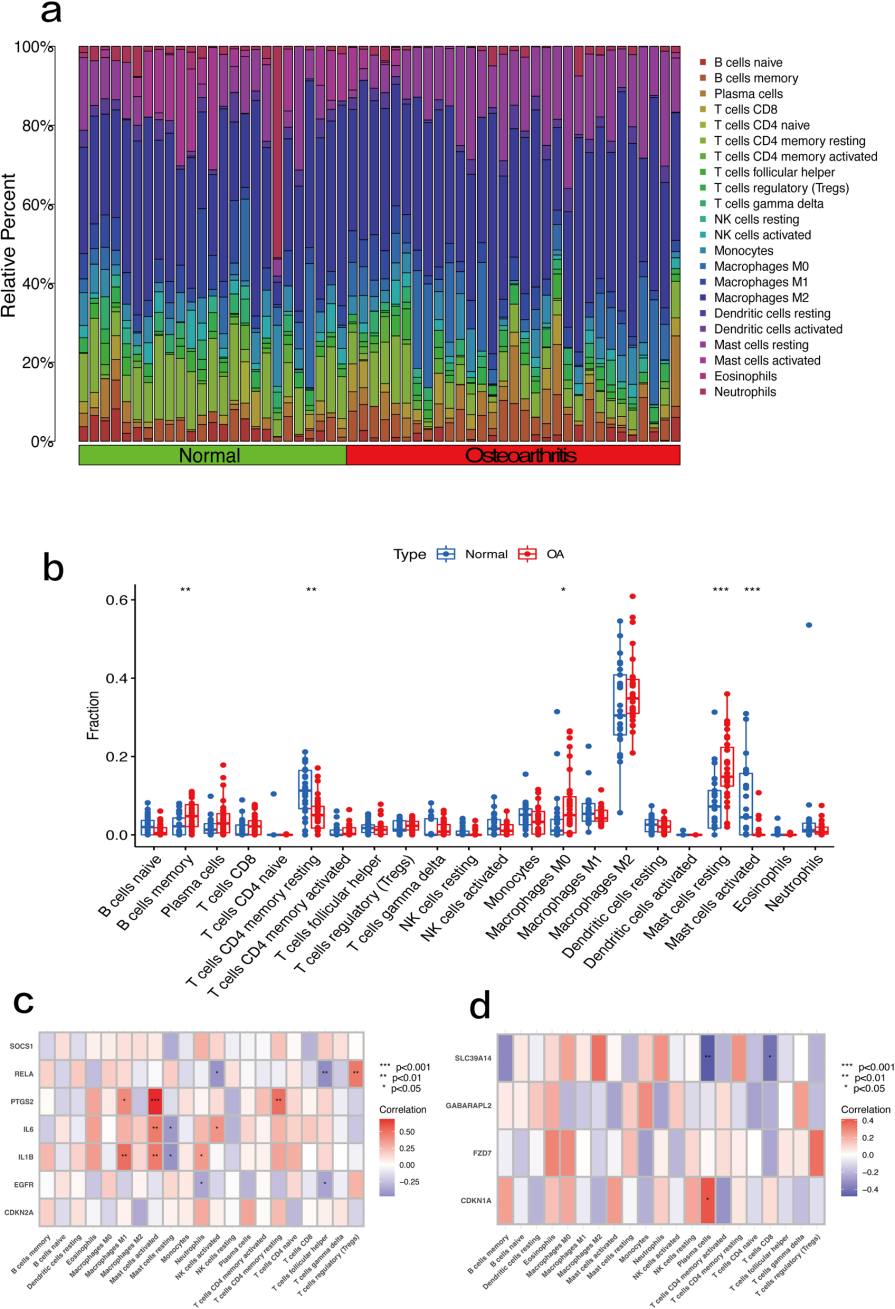
Results of immune infiltration by CIBERSORTx. **a** Bar plot showing the composition of 22 types of immune cells. **b** Box plot presenting the difference of immune infiltration of 22 types of immune cells. Treat represents the OA group, and Control represents the normal group. **c** Heatmap showing the correlation between seven hub genes and 22 types of immune cells in osteoarthritis. **d** Correlation between the four biomarkers and 22 types of immune cells in osteoarthritis

The immune microenvironment plays an important role in the progression of OA. Therefore, with the help of CIBERSORT, we summarized the differences in immune infiltration by immune cell subpopulations between OA samples and non-OA tissues (Fig 9a). The OA samples contained a higher proportion of memory B cells, M0 macrophages, M2 macrophages, and resting mast cells than the control group, as well as a lower proportion of resting CD4 memory T cells and activated mast cells. Correlation analysis showed that activated mast cells showed positive correlations with PTGS2, IL6, and IL1B, and the correlation between activated mast cells and PTGS2 was the highest (0. 686) (Fig. 9b). There were positive correlations between IL1B, PTGS2, and M1 macrophages, resting CD4 memory T cells and PTGS2, and regulatory T cells (Tregs) and RELA. There were significant negative correlations between follicular helper T cells and RELA, as well as between plasma cells and SLC39A14 (Fig. 9c and d).

### Single‑cell analysis

**Fig. 10:**
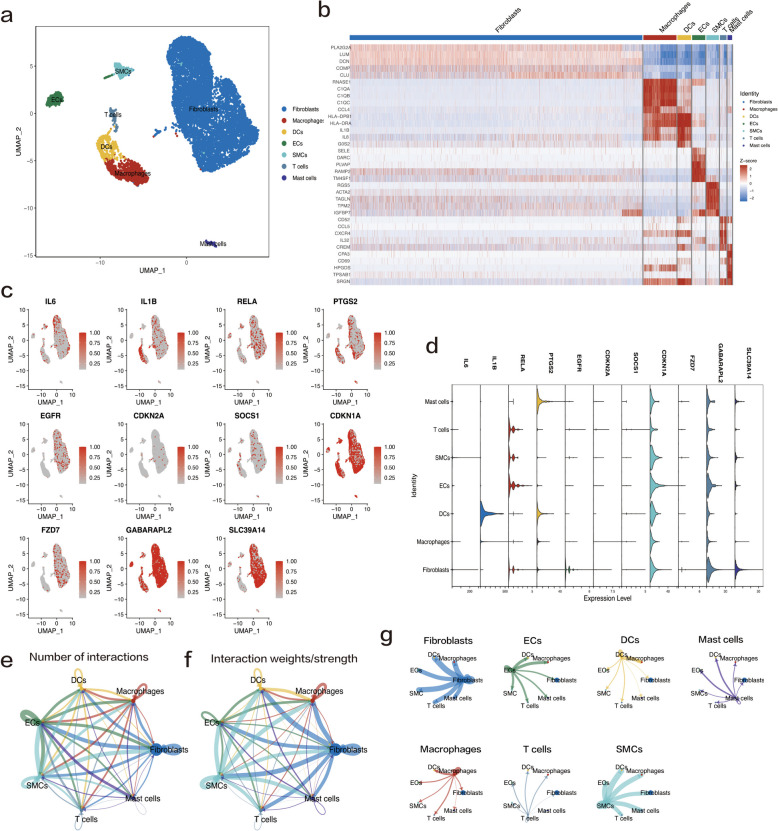
Analysis of single-cell RNA sequencing data from three OA synovial samples. **a** UMAP plot of scRNA-seq showing unsupervised clusters colored according to putative cell types among a total of 10,194 cells in OA synovial samples. The percentages of total acquired cells were as follows: 77.7% fibroblasts, 8.8% macrophages, 3.6% dendritic cells (DCs), 3.5% endothelial cells (ECs), 3.4% smooth muscle cells (SMCs), 1.8% T cells, and 1.2% mast cells. **b** Heatmap depicting the expression levels of the top five marker genes among seven detected cell clusters. **c, d** UMAP plots and violin plots showing the expression of the selected seven hub c-FRGs and four potential biomarkers for each cell type. **e** Interaction net count plot of OA synovial cells. The thicker the line, the greater the number of interactions. **f** Interaction weight plot of synovial cells. The thicker the line, the stronger the interaction weights/strength between the two cell types. **g** Detailed network of cell–cell interactions among seven cell subsets

The scRNA-seq data from three OA synovial samples were obtained from the GSE152805 dataset. After initial quality control, we finally retained 10,194 cells for cell annotation (Supplementary Figure 5). The top 2000 highly variable genes were selected for further analysis (Supplementary Figure 5b). We used the "RunPCA" function to reduce the dimensionality and obtained 14 clusters (Supplementary Figures 6d and e); the first five DEGs of each cluster are shown in Supplementary Table 5. Later, we performed cellular annotation using marker genes and annotated seven cell populations: fibroblasts (77.7%), macrophages (8.8%), dendritic cells (DCs) (3.6%), endothelial cells (ECs) (3.5%), smooth muscle cells (SMCs) (3.4%), T cells (1.8%), and mast cells (1.2%) (Fig. 10a). Next, we performed differential gene expression analysis on these seven cell populations to verify the accuracy of the cell annotation (Fig. 10b). Figures 10c and d show the distribution and expression of seven hub genes and four biomarker genes in different cell populations. We found that 11 c-FRGs were significantly different in macrophages, DCs, mast cells, and NK cells. For example, IL1B, PTGS2, and SLC39A4 were significantly highly expressed in some cells, whereas they were significantly less expressed, or even absent, in other cells. We used CellChat to identify differentially overexpressed ligands and receptors for each cell population. In total, 254 significant ligand–receptor pairs were detected, which were further classified into 62 signaling pathways (Table 2). We found that the immune cells interacted weakly with each other; however, the non-immune cells had extensive communication interactions with other cells and were involved in various paracrine and autocrine signaling interactions (Fig. 10e to g).

**Table 2:** Type and number of ligand–receptor pairs

Communication mode	Number of pathways	Number of L–R pair types	Number of L–R pairs
Cell–cell contact	24	54	312
ECM–receptor	7	121	558
Secreted signaling	31	79	360

*L–R* Ligand–receptor

## Discussion

Copper is an irreplaceable trace metal element that participates in a variety of biological processes. When copper ions accumulate in excess, they eventually lead to cell death, and this new form of programmed cell death is known as cuproptosis [17]. A recent report has demonstrated that copper levels are significantly higher in the serum and synovial tissue of patients with OA than in controls [14]. Evidence from several studies suggests that the development of OA is closely related to ferroptosis in articular cartilage and synovium [25–29], and that OA can be treated to some extent by modulation of ferroptosis [29, 30]. Additionally, previous studies have reported that copper and iron levels are closely correlated with each other in patients with OA [14, 15, 31].

In this study, we identified transcriptional alterations and expression of c-FRGs based on the GSE55235, GSE169077, GSE55457, and GSE55584 datasets. Forty c-FDEGs were identified in 63 c-FRGs. GO enrichment analysis showed that these 40 c-FDEGs were mainly associated with the inflammatory response, cellular response to external stimulus, and autophagy. The KEGG enrichment analysis showed that these genes were highly enriched mainly in the IL-17 signaling pathway, NOD-like receptor signaling pathway, HIF-1 signaling pathway, and TNFα signaling pathway. For both OA and non-OA groups, GSEA and ssGSEA showed that OA was mainly associated with the enrichments in Notch signaling, adipogenesis, xenobiotic metabolism, fatty acid metabolism, peroxisome, TNFα signaling via NF-κB, the inflammatory response, PI3K AKT mTOR signaling, and IL6 JAK STAT3 signaling. This indicates that the mechanism of OA development is closely related to fatty acid metabolism, the inflammatory response, immune regulation, and cell adhesion.

We analyzed the PPI results using the cytoHubba plugin in Cytoscape, revealing seven key c-FDEGs, including IL6, IL1B, RELA, PTGS2, EGFR, CDKN2A, and SOCS1. GSEA and GSVA of the seven genes revealed that IL6, IL1B, RELA, PTGS2, SOCS1, and EGFR were closely associated with inflammation, immune regulation, extracellular matrix, and cell adhesion pathways in OA, which is consistent with previous findings [32, 33]. Interestingly, we also found that they were closely associated with lipid metabolism and fatty acid metabolism in OA. Considering that increased iron accumulation, free radical production, fatty acid supply, and increased lipid peroxidation are key to the induction of ferroptosis [5–7], it is possible that they affect the development of OA by regulating lipid metabolism and fatty acid metabolism, which affects ferroptosis; however, this needs to be further investigated.

Notably, CDKN2A acts as both a cuproptosis-related gene and a ferroptosis-related gene simultaneously. CDKN2A is often considered an important gene in cellular senescence and aging [34], and it is used as a molecular marker of cellular senescence [35]. Our study showed that CDKN2A expression was higher in patients with OA, suggesting that CDKN2A may contribute to the development of OA by affecting cellular senescence and thereby promoting the development of OA.

This is the first study to use the new signature genes combining CRGs with FRGs to reveal the pathogenesis of OA and aid in its treatment. We executed three machine learning algorithms using the 40 c-FDEGs mentioned above and eventually identified four biomarkers: CDKN1A, FZD7, GABARAPL2, and SLC39A14.

Frizzled7 (FZD7) is known to be a receptor of the Wnt pathway. Fzl receptors are usually classified as belonging to the G protein receptor family and are rich in cysteine, which can directly interact with Wnt proteins and thus activate downstream responses [36–38]. Numerous studies have shown that excessive upregulation or downregulation of Wnt signaling pathways in OA may lead to cartilage damage and ultimately accelerate the progression of OA. Therefore, it is necessary and important to maintain a balance in the biological activity of Wnt-related pathways [39–41]. In the present study, FZD7 was significantly increased in the OA group compared with the non-OA group. Therefore, we speculate that an excess of FZD7 may lead to the abnormal activation of Wnt-related pathways and ultimately accelerate the development of OA.

ZIP14 (SLC39A14) is a metal transporter [42] that affects the metabolic balance of zinc, manganese, iron, copper, and other metals [43]. For example, ZIP14 can transport non-transferrin-bound iron (NTBI) [44] and ZIP14 can transport cadmium and manganese through metal/bicarbonate symbiotic activity [45]. It has been shown that OA is closely related to the metabolic balance of metals such as iron, copper, and manganese [14, 15, 31, 46–48]. In this study, we found that ZIP14 was greatly reduced in the OA group compared with the non-OA group. Furthermore, scRNA-seq analysis showed that the distribution of SLC39A14 in OA patients varied significantly among cell populations, with low or even no expression in some cells, which is likely to disrupt the metal metabolic balance in the joints and eventually cause the accumulation of metals such as iron and copper. Therefore, SLC39A14 (ZIP14) may be a very important therapeutic target for OA treatment in the future.

ssGSEA showed that CDKN1A significantly positively correlated with TNF-α signaling via NF-κB, the TGF-β signaling pathway, hypoxia, the P53 pathway, apoptosis, mTORC1 signaling, and other gene sets, suggesting that CDKN1A may affect OA by regulating inflammation, apoptosis, and hypoxia. Although both the CDKN1A and GABARAPL2 genes have been reported previously [49–52], their relationship with ferroptosis and cuproptosis in OA is not yet known. This suggests that these genes may be targets not only for immunotherapy, inflammation, and autophagy but also for the treatment of cuproptosis and ferroptosis in OA. Notably, we found that melphalan, paclitaxel, vinblastine, and vantictumab may serve as potential drugs for the treatment of OA. Previous studies have reported that they act therapeutically by regulating CDKN1A or FZD7 [53–55], thus affecting processes such as the cell cycle, cell proliferation, and apoptosis, which also validates our prediction. We then constructed a disease model of OA based on these four biomarkers that could significantly improve our ability to recognize OA at an early stage. Thus, our findings suggest that CDKN1A, FZD7, GABARAPL2, and SLC39A14 are excellent disease biomarkers and potential therapeutic targets for OA, and the disease model constructed based on them has good diagnostic efficacy.

Recently, an increasing number of studies have shown that immune cell infiltration is essential for OA onset and development and cartilage repair [56–58]. Our study showed a close relationship between the seven hub genes and immune cells. Notably, there were significant positive correlations of PTGS2, IL6, and IL1B with M1 macrophages and activated mast cells. Previous studies have demonstrated that the activation of macrophages and mast cells may significantly accelerate the progression of OA [58–60]. Therefore, we speculate that PTGS2, IL6, and IL1B may influence the onset and progression of OA by regulating these cells. Interestingly, scRNA-seq analysis further revealed that PTGS2 was significantly highly expressed in mast cells, leading us to speculate that PTGC2 may influence the progression of OA by regulating the activation of mast cells and thus the progression of OA. Surprisingly, we found weak interactions between immune cells in the synovial tissue of patients with OA, whereas there were complex communication networks between immune and non-immune cells (fibroblasts, SMCs, and ECs). These hypotheses and questions require more studies to reveal intricate interrelationships between these c-FRGs, immune cells, and OA.

In addition, we found that C10orf91 could regulate CDKN1A and SLC39A14 by regulating hsa-miR-149-3p, hsa-miR-423-5p, hsa-miR-31-5p, and hsa-miR-30b-3p. Both hsa-miR-513a-3p and has-miR-548c-3p can regulate both CDKN1A and GABARAPL2; however, no related study has been reported yet, so this needs to be further investigated and validated in the future.

This study was conducted mainly using bioinformatics analysis, and despite the combination of scRNA-seq analysis and the use of powerful machine learning algorithms, such as RF and SVM-RFE, there are still some limitations to our study. First, the small sample size of the analysis may have led to inaccuracies in the determination of hub genes, CIBERSORT analysis, and single-cell analysis. Second, although the disease model nomogram was well validated, the data was obtained retrospectively from public databases, meaning that inherent selection bias may have affected their accuracy. In addition, while our data can show the correlation between OA and immune cells, they cannot reveal causality. Extensive prospective studies, as well as complementary in vivo and in vitro experimental studies, are necessary to validate the accuracy of potential therapeutic targets and biomarkers.

## Conclusions

Our study showed that four genes—CDKN1A, FZD7, GABARAPL2, and SLC39A14—are good disease biomarkers and potential therapeutic targets for OA. Our study provides a theoretical basis and research direction for understanding the role of c-FRGs in the pathophysiological process and for potential therapeutic intervention in OA.

### Supplementary Information

**Supplementary Material 1.**

## Data Availability

The datasets used or analysed during the current study are available from the corresponding author on reasonable request.

## Abbreviations

OA: Osteoarthritis
NSAIDs: Nonsteroidal anti-inflammatory drugs
ROS: Reactive oxygen species
FRGs: Ferroptosis-related genes
TCA: Tricarboxylic acid
CRGs: Cuproptosis-related genes
FRGs: Ferroptosis-related genes
c-FRGs: The new signature genes combining cuproptosis-related genes (CRGs) with ferroptosis-related genes (FRGs)
NCBI: National Center for Biotechnology Information
GEO: Gene expression omnibus
DEGs: Differentially expressed genes
c-FDEGs: Differentially expressed c-FRGs
GO: Gene Ontology
KEGG: Kyoto Encyclopedia of Genes and Genomes
GSEA: Gene set enrichment analysis
SVM-RFE: Support vector machine recursive feature elimination
RF: Random forest analysis
LASSO: Least absolute shrinkage and selection operator
ROC: Receiver operating characteristic
