# Supplementary material for: Identification and verification of a novel signature that combines cuproptosis-related genes with ferroptosis-related genes in osteoarthritis using bioinformatics analysis and experimental validation
Source: Arthritis Res Ther. 2024 May 13;26:100. doi: 10.1186/s13075-024-03328-3 (PMC11089679; doi:10.1186/s13075-024-03328-3)
Supplement: Supplementary file 1 — Supplementary Material 1. [file 13075_2024_3328_MOESM1_ESM.docx]

**Supplementary files:**

**** **Supplementary Figure 1:** Correlation between the 40 differentially expressed the novel signature genes(c-FRGs).

**
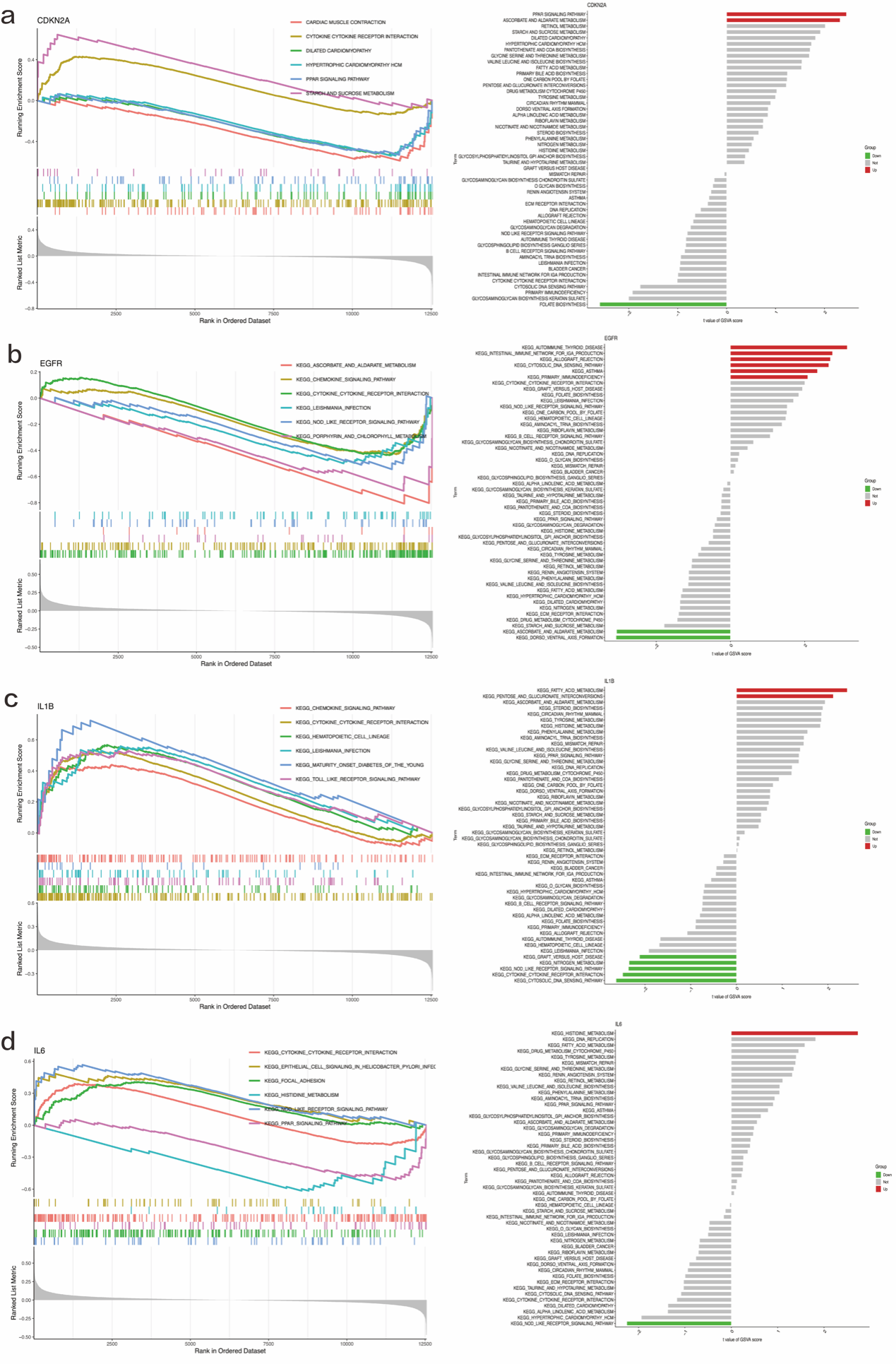
**


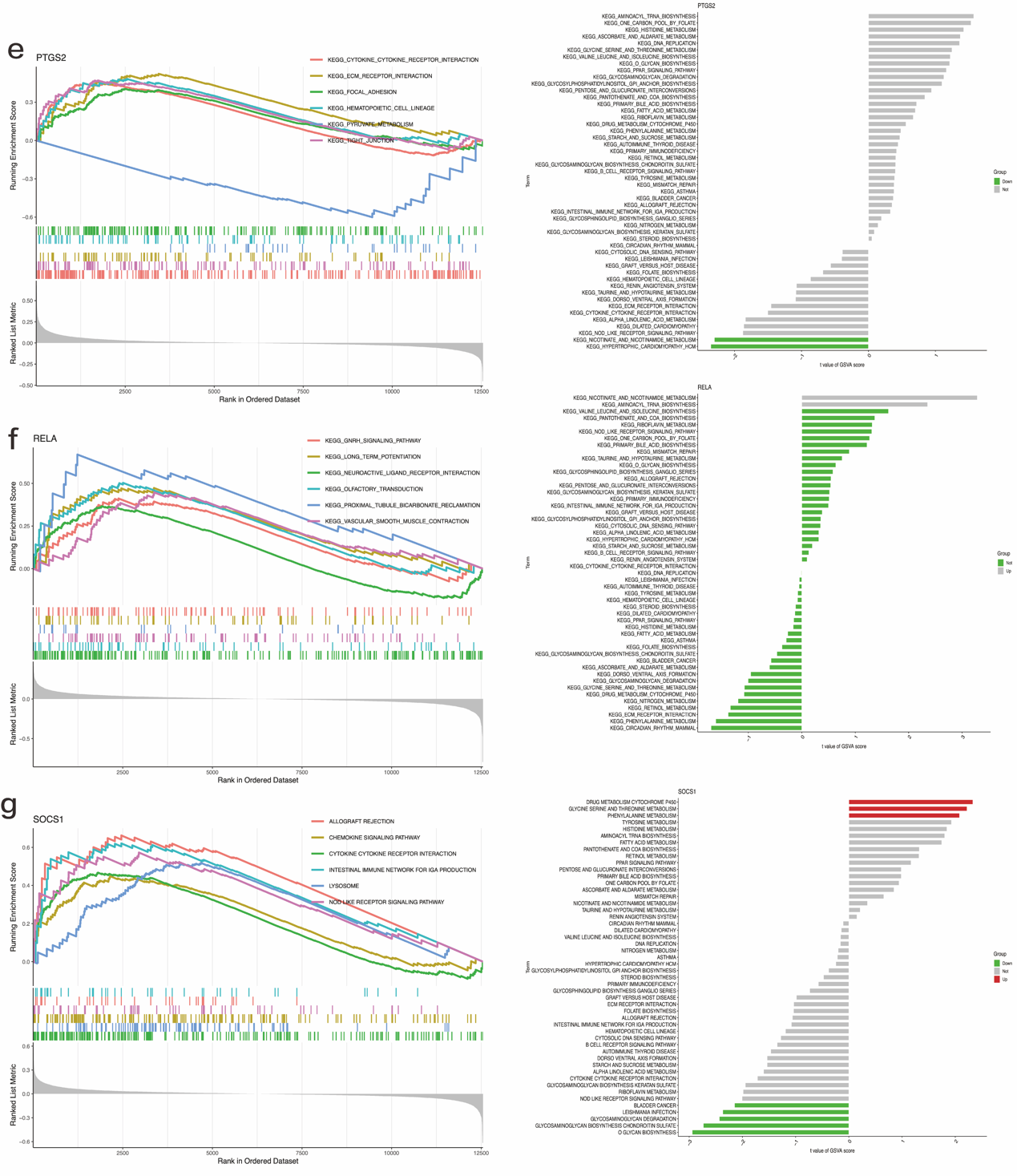


**Supplementary Figure 2:** (a–g) Single-gene GSEA-KEGG and single-gene GSVA results of seven hub genes.

**
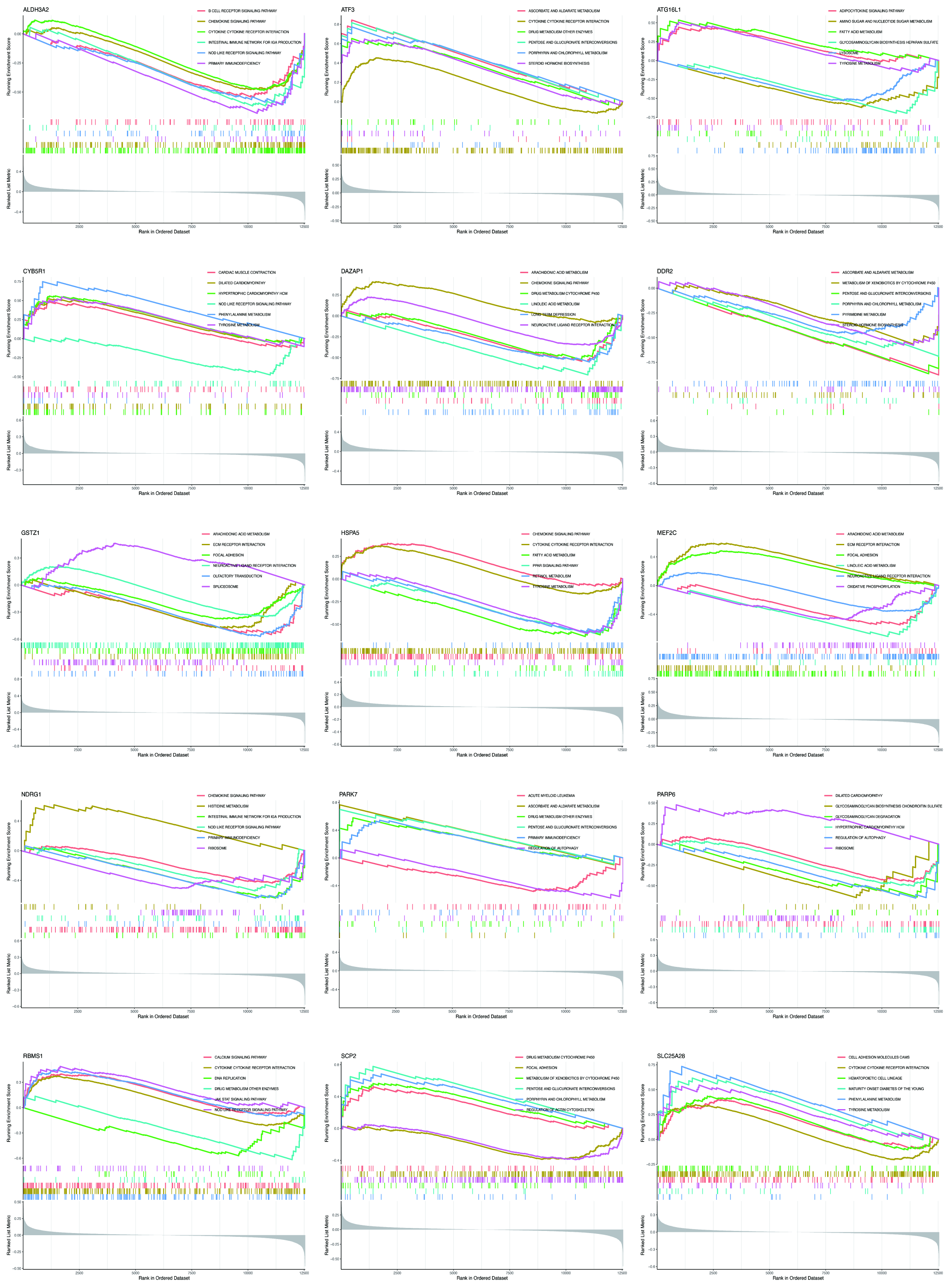
**

**
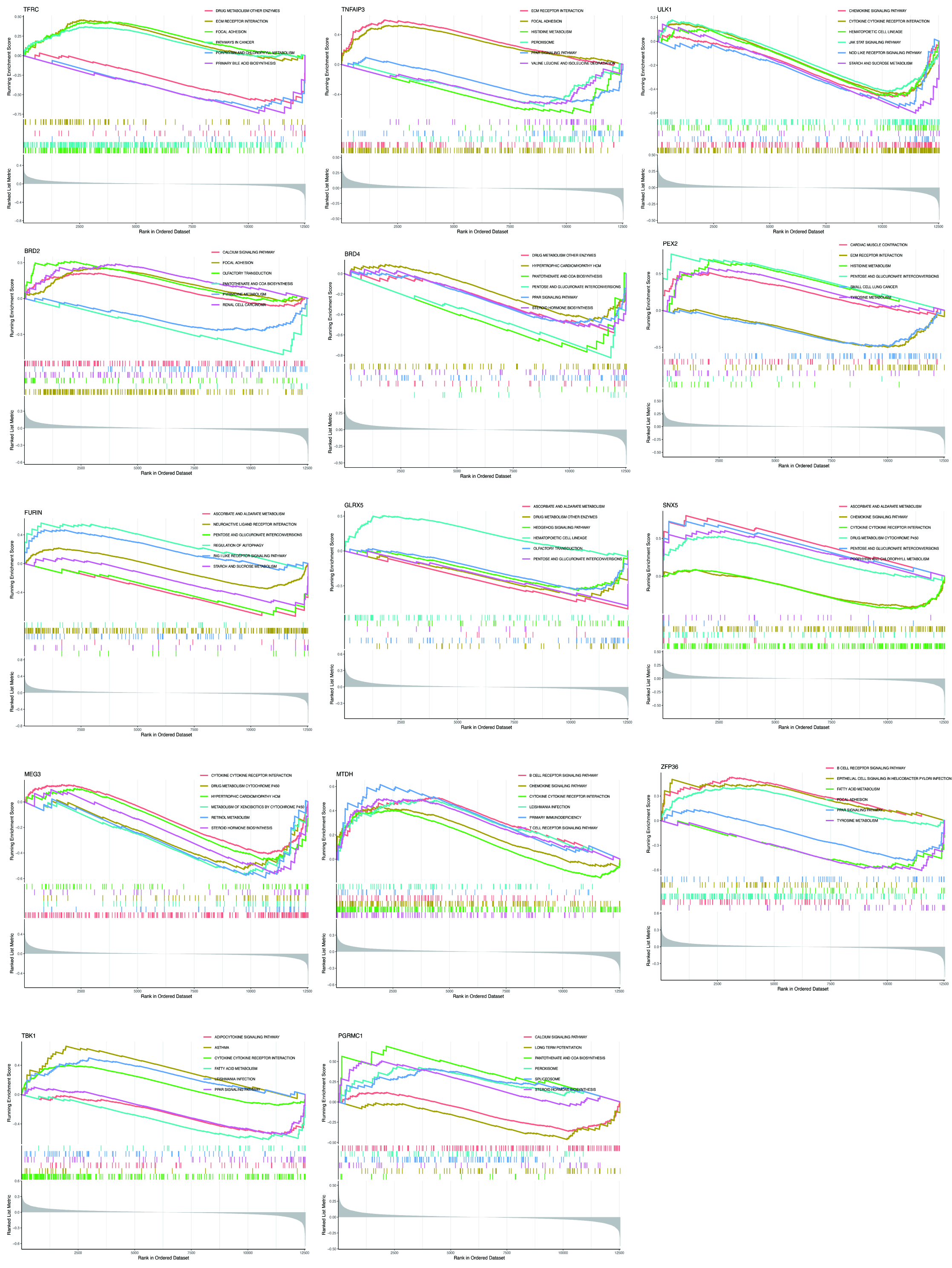
**

**Supplementary Figure 3:** Single-gene GSEA-KEGG pathway analysis of the additional 29 differentially expressed c-FRGs. We show the top six pathways with the smallest p-value.

**
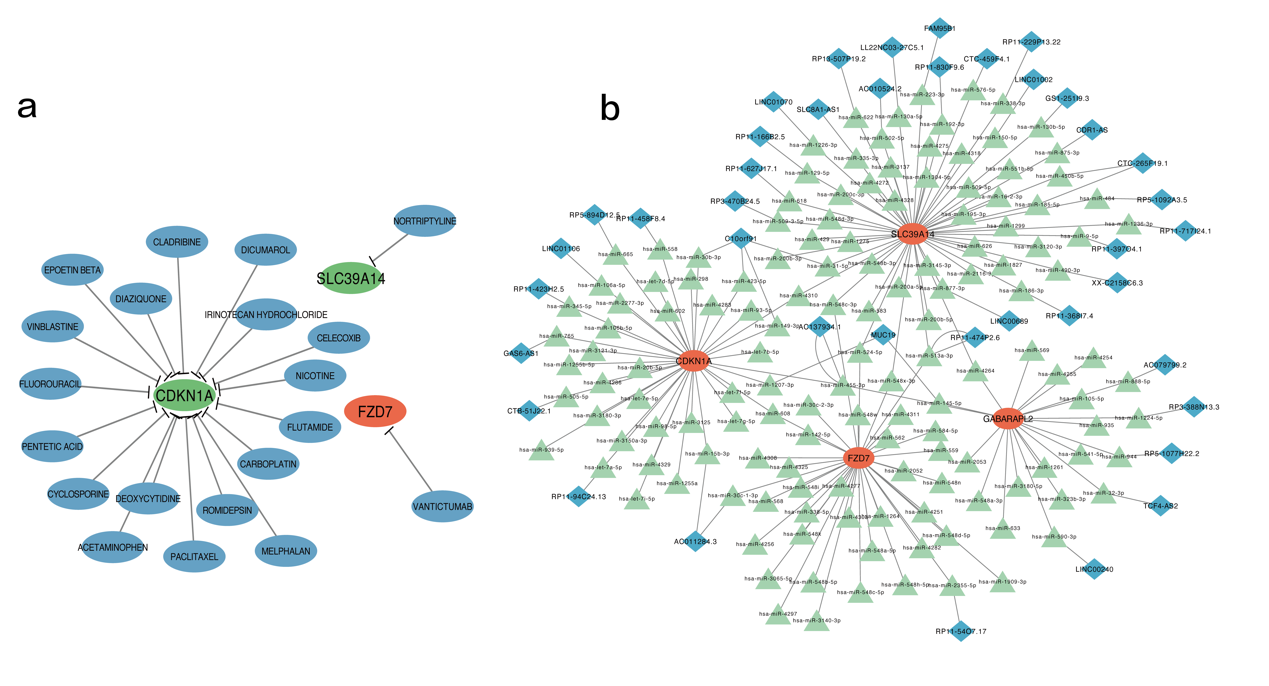
**

**Supplementary Figure 4:** Construction of drug prediction network and ceRNA network. (a) Drug prediction network constructed based on four biomarkers. The upregulated biomarkers are represented by red ellipses, the downregulated biomarkers are indicated by green ellipses, and the blue ellipses represent drugs predicted based on the biomarkers. (b) ceRNA network constructed based on four biomarkers. The diamond represents lncRNAs, the triangle represents miRNAs, and the circle represents mRNAs.


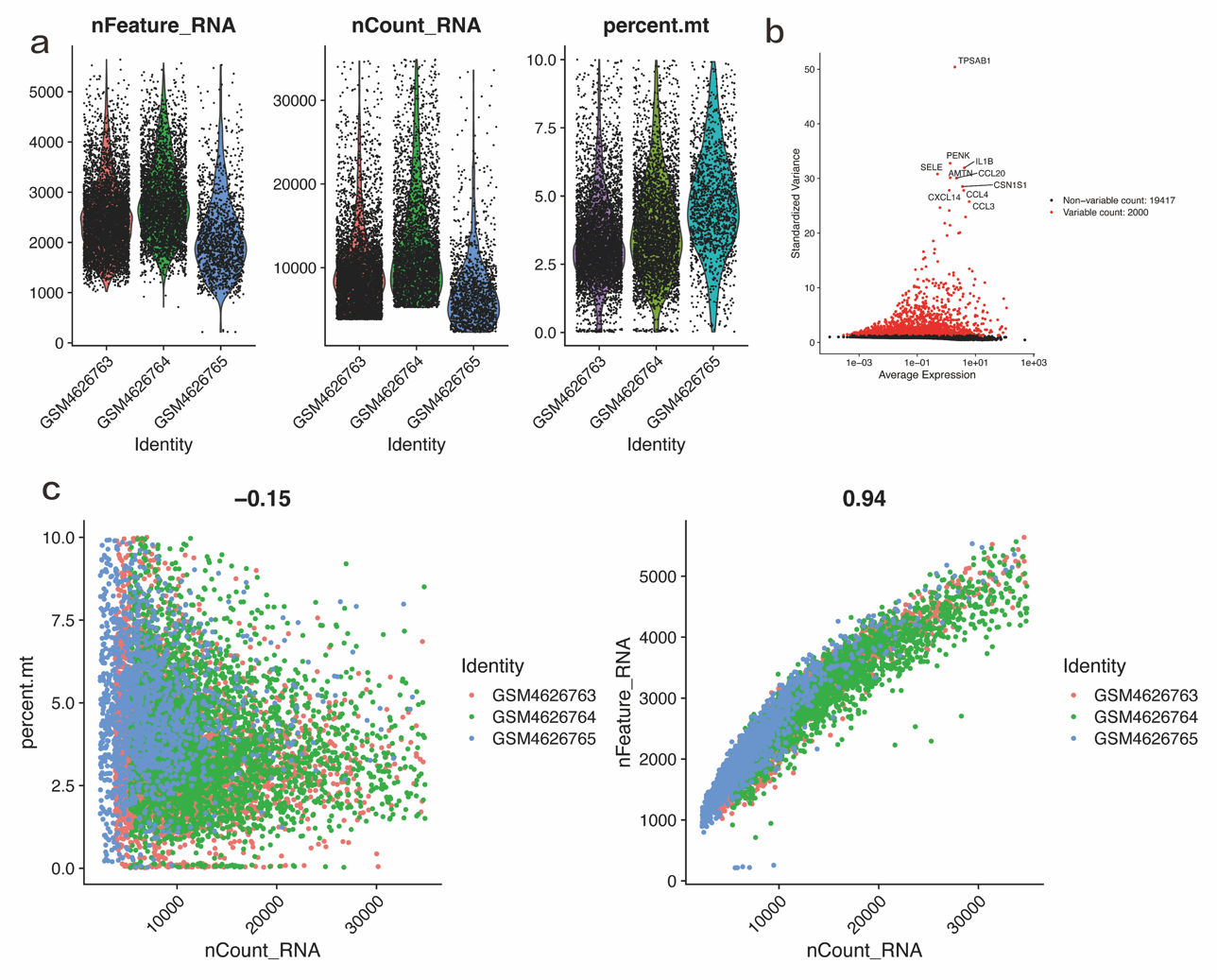


**Supplementary Figure 5: Characterization of single-cell RNA sequencing from human synovium OA environments.** (a) Quality control of scRNA-seq of synovium samples from three OA patients to filter out the cells with poor quality. (b) We identified the highly variable genes across the cells and drew the characteristic variance diagram. (c) We analyzed the correlations between the detected gene counts and sequencing depth.


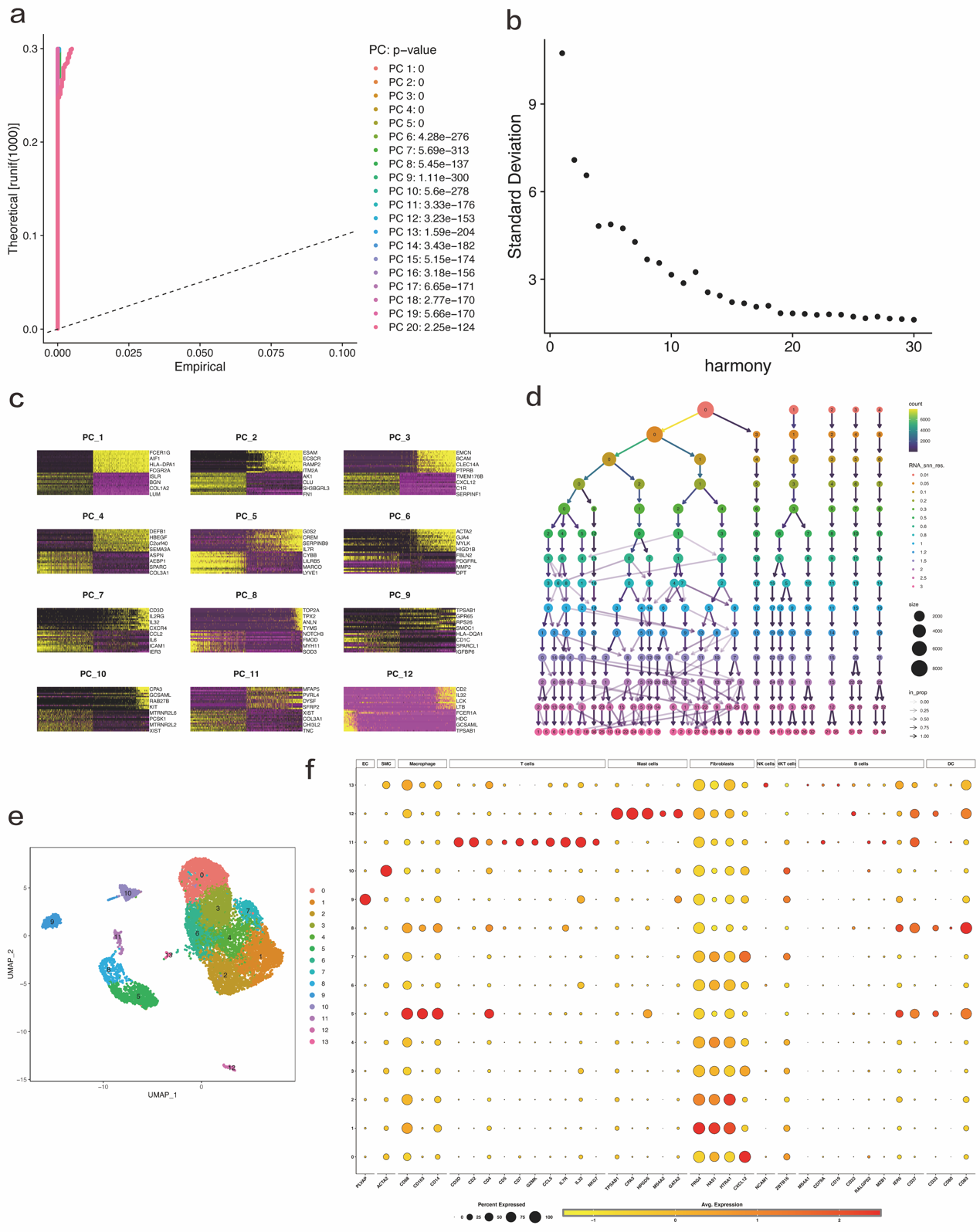


**Supplementary Figure 6: The significantly correlated genes in the top 12 PCs and cellular annotation of scRNA-seq data.** (a) The top 20 principal components were identified based on p value < 0.05. (b) We drew the elbow plot to determine the optimal PC number. (c) Heat map showing the related gene expression in the top six PCs. (d) Sankey diagram showing the cell clustering at 13 different resolutions (resolution = 0.01, 0.05, 0.1, 0.2, 0.3, 0.6, 0.8, 1, 1.2, 1.5, 2, 2.5, 3). (e) Cells were divided into 14 separate clusters by “RunUMAP” function. (f) We annotated the cells according to the cell-specific marker genes.


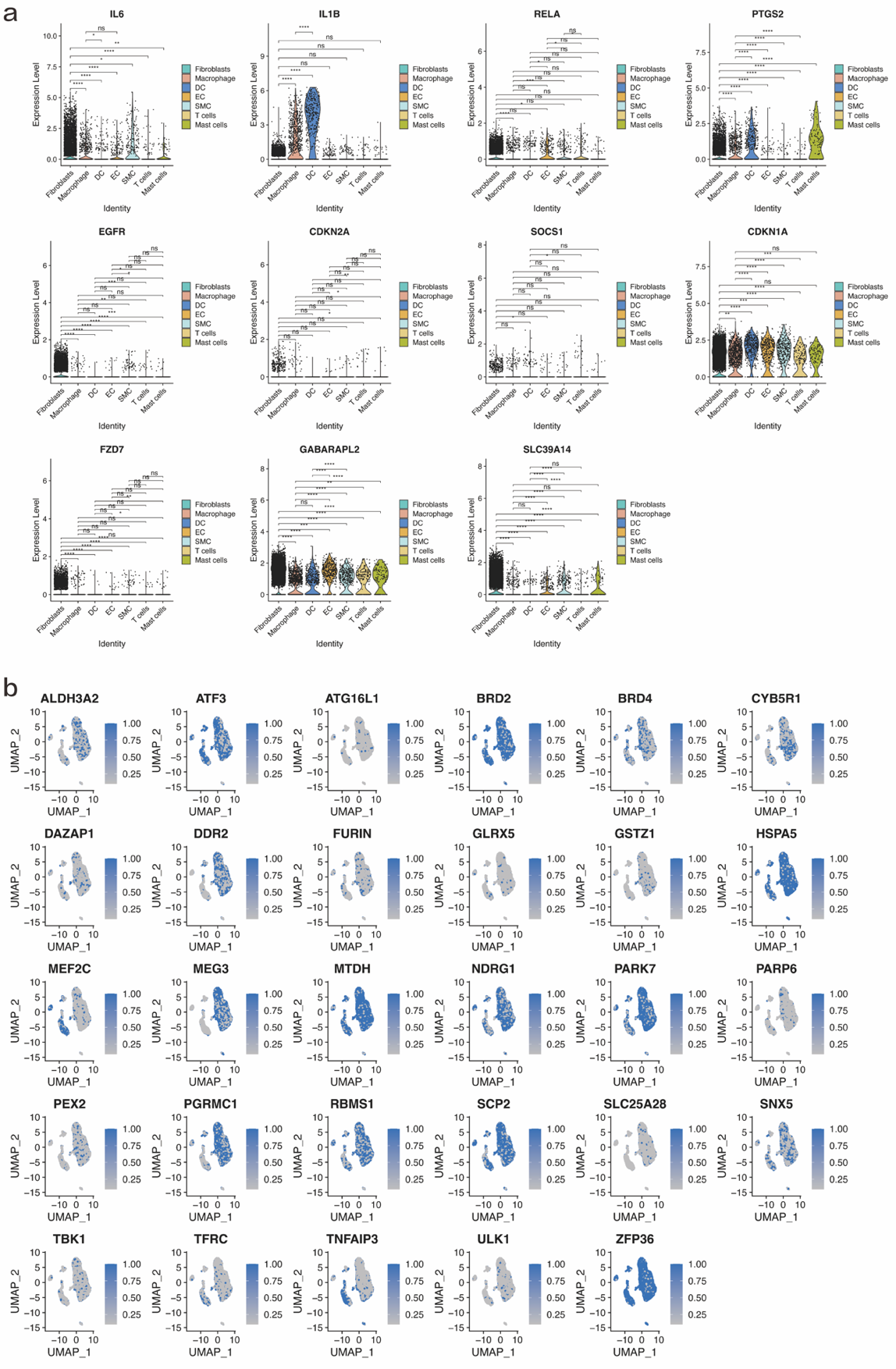


**Supplementary Figure 7:** **Distribution of** **differentially expressed c-FRGs in OA based on single-cell RNA sequencing data.** (a) Violin plots show the differential expression of seven hub genes and four potential biomarkers in different cell types. ^ns^p > 0.05, *p < 0.05, **p < 0.01, ***p < 0.001, ****p < 0.0001. (b) Expression of the remaining 29 differentially expressed c-FRGs for each cell population is shown in UMAP plots.

**Supplementary Table 1:** A total of 81 particular FRGs (c-FRGs) were highly correlated with 11 CRGs (|r| > 0.5, adj. p value < 0.05).

| CRG | FRG | cor | p | Regulation |
| --- | --- | --- | --- | --- |
| LIPT1 | ALDH3A2 | 0.566143816 | 4.43E-06 | postive |
| MTF1 | AMN | 0.545329204 | 1.15E-05 | postive |
| DLAT | ATF2 | 0.551116104 | 8.89E-06 | postive |
| CDKN2A | ATF3 | -0.507636819 | 5.57E-05 | negative |
| DBT | ATG16L1 | 0.554129472 | 7.75E-06 | postive |
| DLAT | ATG5 | 0.514146955 | 4.30E-05 | postive |
| FDX1 | BRD2 | -0.576721731 | 2.66E-06 | negative |
| PDHB | BRD2 | -0.521197012 | 3.23E-05 | negative |
| FDX1 | BRD4 | -0.507823163 | 5.52E-05 | negative |
| MTF1 | CD44 | 0.506284612 | 5.87E-05 | postive |
| PDHB | CD44 | -0.579941175 | 2.27E-06 | negative |
| LIPT1 | CDKN1A | -0.558246456 | 6.42E-06 | negative |
| PDHB | CDKN1A | -0.608040165 | 5.26E-07 | negative |
| CDKN2A | CDKN2A | 1 | 0 | postive |
| DLD | CDO1 | 0.502311539 | 6.85E-05 | postive |
| MTF1 | CHMP5 | -0.523578038 | 2.92E-05 | negative |
| DBT | CYB5R1 | -0.51865976 | 3.58E-05 | negative |
| LIPT1 | DAZAP1 | -0.551667421 | 8.67E-06 | negative |
| PDHB | DAZAP1 | -0.594410295 | 1.09E-06 | negative |
| FDX1 | DDR2 | -0.598556134 | 8.75E-07 | negative |
| DLAT | DLD | 0.52185857 | 3.14E-05 | postive |
| DLD | DLD | 1 | 0 | postive |
| FDX1 | EGFR | -0.568320524 | 4.00E-06 | negative |
| FDX1 | EMC2 | 0.529330702 | 2.30E-05 | postive |
| NFE2L2 | ETV4 | -0.502191139 | 6.88E-05 | negative |
| MTF1 | FURIN | 0.566139409 | 4.44E-06 | postive |
| PDHB | FURIN | -0.506046534 | 5.92E-05 | negative |
| PDHB | FZD7 | 0.56957363 | 3.77E-06 | postive |
| LIPT1 | GABARAPL2 | 0.532516187 | 2.01E-05 | postive |
| PDHB | GABARAPL2 | 0.651260048 | 4.12E-08 | postive |
| DLAT | GCLC | 0.516593204 | 3.89E-05 | postive |
| LIPT1 | GLRX5 | 0.565696418 | 4.53E-06 | postive |
| PDHB | GLRX5 | 0.539824194 | 1.47E-05 | postive |
| PDHB | GSTZ1 | 0.528692524 | 2.36E-05 | postive |
| LIPT1 | HIF1A | -0.641122515 | 7.76E-08 | negative |
| DBT | HMGB1 | 0.524328457 | 2.84E-05 | postive |
| LIPT1 | HMOX1 | -0.518019661 | 3.67E-05 | negative |
| LIPT1 | HSPA5 | -0.554690735 | 7.56E-06 | negative |
| LIPT1 | IL1B | -0.559683943 | 6.00E-06 | negative |
| LIPT1 | IL6 | -0.603780169 | 6.63E-07 | negative |
| LIPT1 | KDM6B | -0.676128829 | 7.85E-09 | negative |
| PDHB | KDM6B | -0.612406285 | 4.14E-07 | negative |
| PDHA1 | LYRM1 | 0.500406961 | 7.37E-05 | postive |
| PDHB | MEF2C | 0.506079643 | 5.92E-05 | postive |
| DBT | MEG3 | 0.501112506 | 7.18E-05 | postive |
| MTF1 | MTDH | 0.587222314 | 1.57E-06 | postive |
| GLS | MTF1 | 0.506397721 | 5.84E-05 | postive |
| MTF1 | MTF1 | 1 | 0 | postive |
| PDHB | MTF1 | -0.502262141 | 6.86E-05 | negative |
| PDHB | NDRG1 | -0.583513574 | 1.90E-06 | negative |
| NFE2L2 | NFE2L2 | 1 | 0 | postive |
| LIPT1 | OSBPL9 | 0.505485548 | 6.06E-05 | postive |
| MTF1 | PARK7 | -0.601405325 | 7.52E-07 | negative |
| DBT | PARP6 | 0.691541553 | 2.59E-09 | postive |
| PDHB | PARP6 | -0.549481213 | 9.57E-06 | negative |
| LIPT1 | PEX2 | 0.574328388 | 2.99E-06 | postive |
| DLAT | PGRMC1 | 0.572690748 | 3.24E-06 | postive |
| PDHB | PGRMC1 | 0.562500394 | 5.27E-06 | postive |
| LIPT1 | PTGS2 | -0.626889934 | 1.82E-07 | negative |
| DLAT | RB1 | 0.529350198 | 2.30E-05 | postive |
| LIPT1 | RBMS1 | -0.558475038 | 6.35E-06 | negative |
| MTF1 | RBMS1 | 0.685587919 | 4.00E-09 | postive |
| LIPT1 | RELA | -0.506589525 | 5.80E-05 | negative |
| PDHB | RELA | -0.556243873 | 7.04E-06 | negative |
| GLS | RNF113A | -0.638636719 | 9.03E-08 | negative |
| MTF1 | RNF113A | -0.537486656 | 1.62E-05 | negative |
| FDX1 | SCP2 | 0.553267348 | 8.06E-06 | postive |
| PDHB | SCP2 | 0.627455926 | 1.76E-07 | postive |
| PDHB | SLC25A28 | -0.578658903 | 2.42E-06 | negative |
| LIPT1 | SLC39A14 | -0.533019263 | 1.97E-05 | negative |
| LIPT1 | SMAD7 | -0.555924946 | 7.14E-06 | negative |
| LIPT1 | SNX5 | 0.538811945 | 1.53E-05 | postive |
| LIPT1 | SOCS1 | -0.622697836 | 2.32E-07 | negative |
| MTF1 | SREBF2 | 0.548186111 | 1.01E-05 | postive |
| LIPT1 | TBK1 | -0.595676148 | 1.02E-06 | negative |
| LIPT1 | TFRC | -0.58768925 | 1.54E-06 | negative |
| LIPT1 | TNFAIP3 | -0.567593712 | 4.14E-06 | negative |
| NFE2L2 | TTPA | 0.517967037 | 3.68E-05 | postive |
| FDX1 | ULK1 | -0.540732186 | 1.41E-05 | negative |
| LIPT1 | ZFP36 | -0.551451518 | 8.76E-06 | negative |

FRG, cuproptosis-related gene.

CRG, ferroptosis-related gene.

**Supplementary Table 2:** Detailed information about the 40 differentially expressed c-FRGs.

| Gene | conMean | treatMean | pvalue | Type |
| --- | --- | --- | --- | --- |
| ALDH3A2 | 9.08476496 | 9.473631969 | 0.003085604 | Up |
| ATF3 | 10.1653904 | 8.169401531 | 6.22E-09 | Down |
| ATG16L1 | 5.28535608 | 4.541472219 | 0.011051096 | Down |
| BRD2 | 10.13284132 | 9.570883438 | 7.61E-07 | Down |
| BRD4 | 9.2571424 | 8.629470281 | 0.00014671 | Down |
| CDKN1A | 10.52573828 | 9.005631656 | 5.75E-10 | Down |
| CDKN2A | 6.4018678 | 6.798183188 | 0.000759491 | Up |
| CYB5R1 | 9.93277248 | 10.32750766 | 1.02E-05 | Up |
| DAZAP1 | 8.32334328 | 7.893812625 | 4.30E-05 | Down |
| DDR2 | 9.71976844 | 9.293857656 | 0.024044973 | Down |
| EGFR | 7.73859136 | 7.351112594 | 0.00226628 | Down |
| FURIN | 8.60808504 | 8.076414344 | 2.63E-05 | Down |
| FZD7 | 8.41904468 | 9.165387688 | 1.64E-07 | Up |
| GABARAPL2 | 11.27119732 | 11.63532956 | 1.73E-08 | Up |
| GLRX5 | 8.54478588 | 8.993341 | 0.002400324 | Up |
| GSTZ1 | 5.13093688 | 6.094834281 | 0.001903565 | Up |
| HSPA5 | 11.88396968 | 11.33786566 | 0.001252066 | Down |
| IL1B | 7.83665464 | 6.981765656 | 0.00336501 | Down |
| IL6 | 8.81210256 | 7.026818563 | 0.00014671 | Down |
| MEF2C | 8.51681548 | 8.846308969 | 0.018448404 | Up |
| MEG3 | 7.8299334 | 7.237481719 | 0.001794836 | Down |
| MTDH | 9.78339376 | 9.627593281 | 0.032343396 | Down |
| NDRG1 | 11.59299096 | 11.09523409 | 0.000279075 | Down |
| PARK7 | 11.93305964 | 12.12019769 | 0.000670583 | Up |
| PARP6 | 9.49148084 | 9.140185906 | 1.04E-06 | Down |
| PEX2 | 8.6058074 | 9.04399275 | 5.61E-05 | Up |
| PGRMC1 | 9.94748532 | 10.27102344 | 1.36E-05 | Up |
| PTGS2 | 7.6265094 | 6.062853531 | 0.000343303 | Down |
| RBMS1 | 11.02747684 | 10.8149125 | 0.007444185 | Down |
| RELA | 9.7972514 | 9.465734594 | 0.000142735 | Down |
| SCP2 | 9.84915228 | 10.25453706 | 3.22E-07 | Up |
| SLC25A28 | 8.85529224 | 8.239892875 | 9.17E-08 | Down |
| SLC39A14 | 12.05687724 | 11.07607966 | 1.90E-06 | Down |
| SNX5 | 9.54522528 | 9.792168875 | 0.0014509 | Up |
| SOCS1 | 5.7987278 | 5.322698594 | 0.000109863 | Down |
| TBK1 | 9.09316312 | 8.782777969 | 2.23E-05 | Down |
| TFRC | 9.82407924 | 9.147238625 | 0.003966707 | Down |
| TNFAIP3 | 9.59694536 | 8.421950406 | 1.70E-09 | Down |
| ULK1 | 7.2431838 | 6.7959855 | 0.005758126 | Down |
| ZFP36 | 12.16774056 | 10.61200656 | 5.71E-08 | Down |

c-FRGs, particular FRGs highly correlated with 11 CRGs (|r|>0.5, adj. p value <0.05).

**Supplementary Table 3:** Description of Gene Ontology terms in Figure 3a.

| ID | Description | GeneRatio | p.adjust | geneID |
| --- | --- | --- | --- | --- |
| BP | regulation of inflammatory response | 10/40 | 1.28E-05 | BRD4/EGFR/FURIN/IL1B/IL6/PARK7/PTGS2/RELA/TNFAIP3/ZFP36 |
|  | positive regulation of cellular catabolic process | 10/40 | 1.67E-05 | ATG16L1/FURIN/IL1B/IL6/MTDH/PARK7/TBK1/TNFAIP3/ULK1/ZFP36 |
|  | positive regulation of catabolic process | 10/40 | 3.18E-05 | ATG16L1/FURIN/IL1B/IL6/MTDH/PARK7/TBK1/TNFAIP3/ULK1/ZFP36 |
|  | regulation of DNA-binding transcription factor activity | 9/40 | 8.25E-05 | CDKN2A/DDR2/IL1B/IL6/MTDH/PARK7/RELA/TFRC/TNFAIP3 |
|  | response to nutrient levels | 9/40 | 9.42E-05 | ATF3/CDKN1A/EGFR/GABARAPL2/HSPA5/PTGS2/RELA/ULK1/ZFP36 |
|  | cellular response to biotic stimulus | 8/40 | 3.00E-05 | HSPA5/IL1B/IL6/MEF2C/MTDH/RELA/TNFAIP3/ZFP36 |
|  | cellular response to external stimulus | 8/40 | 8.25E-05 | ATF3/CDKN1A/EGFR/GABARAPL2/HSPA5/IL1B/PTGS2/ULK1 |
|  | response to lipopolysaccharide | 8/40 | 9.42E-05 | IL1B/IL6/MEF2C/MTDH/PTGS2/RELA/TNFAIP3/ZFP36 |
|  | response to molecule of bacterial origin | 8/40 | 0.000122682 | IL1B/IL6/MEF2C/MTDH/PTGS2/RELA/TNFAIP3/ZFP36 |
|  | response to xenobiotic stimulus | 8/40 | 0.000340277 | CDKN1A/EGFR/HSPA5/IL1B/MEF2C/PTGS2/RELA/TFRC |
| CC | autophagosome membrane | 3/39 | 0.009164199 | ATG16L1/GABARAPL2/ULK1 |
|  | phagophore assembly site membrane | 2/39 | 0.026632618 | ATG16L1/ULK1 |
|  | recycling endosome | 4/39 | 0.026632618 | FZD7/NDRG1/TFRC/ULK1 |
|  | autophagosome | 3/39 | 0.034058814 | ATG16L1/GABARAPL2/ULK1 |
|  | transferase complex, transferring phosphorus-containing groups | 4/39 | 0.034058814 | CDKN1A/PEX2/SOCS1/ULK1 |
|  | phagophore assembly site | 2/39 | 0.037410706 | ATG16L1/ULK1 |
|  | smooth endoplasmic reticulum | 2/39 | 0.037410706 | HSPA5/PGRMC1 |
|  | peroxisome | 3/39 | 0.041844195 | ALDH3A2/PEX2/SCP2 |
|  | microbody | 3/39 | 0.041844195 | ALDH3A2/PEX2/SCP2 |
|  | membrane raft | 4/39 | 0.050398447 | EGFR/FURIN/PARK7/PTGS2 |
| MF | NF-kappaB binding | 3/39 | 0.00700301 | CDKN2A/MTDH/RELA |
|  | oxidoreductase activity, acting on peroxide as acceptor | 3/39 | 0.01672261 | GSTZ1/PARK7/PTGS2 |
|  | cyclin-dependent protein serine/threonine kinase inhibitor activity | 2/39 | 0.01672261 | CDKN1A/CDKN2A |
|  | ubiquitin protein ligase binding | 5/39 | 0.01672261 | CDKN1A/EGFR/GABARAPL2/HSPA5/RELA |
|  | protein kinase inhibitor activity | 3/39 | 0.01672261 | CDKN1A/CDKN2A/SOCS1 |
|  | kinase inhibitor activity | 3/39 | 0.01672261 | CDKN1A/CDKN2A/SOCS1 |
|  | ubiquitin-like protein ligase binding | 5/39 | 0.01672261 | CDKN1A/EGFR/GABARAPL2/HSPA5/RELA |
|  | cadherin binding | 5/39 | 0.01830243 | EGFR/HSPA5/NDRG1/PARK7/SNX5 |
|  | antioxidant activity | 3/39 | 0.01839417 | GSTZ1/PARK7/PTGS2 |
|  | lysine-acetylated histone binding | 2/39 | 0.01839417 | BRD2/BRD4 |

**Supplementary Table 4:** Description of Kyoto Encyclopedia of Genes and Genomes terms in Figure 3b.

| ID | Description | GeneRatio | p.adjust | geneID |
| --- | --- | --- | --- | --- |
| hsa05022 | Pathways of neurodegeneration - multiple diseases | 9/28 | 0.000798140276106385 | FZD7/HSPA5/IL1B/IL6/PARK7/PTGS2/RELA/TBK1/ULK1 |
| hsa05163 | Human cytomegalovirus infection | 8/28 | 5.32368440317849E-05 | CDKN1A/CDKN2A/EGFR/IL1B/IL6/PTGS2/RELA/TBK1 |
| hsa04621 | NOD-like receptor signaling pathway | 7/28 | 0.000121464828905529 | ATG16L1/GABARAPL2/IL1B/IL6/RELA/TBK1/TNFAIP3 |
| hsa05010 | Alzheimer disease | 7/28 | 0.00301450854937655 | FZD7/IL1B/IL6/PTGS2/RELA/SLC39A14/ULK1 |
| hsa04657 | IL-17 signaling pathway | 6/28 | 5.32368440317849E-05 | IL1B/IL6/PTGS2/RELA/TBK1/TNFAIP3 |
| hsa05167 | Kaposi sarcoma-associated herpesvirus infection | 6/28 | 0.0010139421554796 | CDKN1A/IL6/PTGS2/RELA/TBK1/ZFP36 |
| hsa05165 | Human papillomavirus infection | 6/28 | 0.00625131977853114 | CDKN1A/EGFR/FZD7/PTGS2/RELA/TBK1 |
| hsa04066 | HIF-1 signaling pathway | 5/28 | 0.000991919445870356 | CDKN1A/EGFR/IL6/RELA/TFRC |
| hsa04668 | TNF signaling pathway | 5/28 | 0.000991919445870356 | IL1B/IL6/PTGS2/RELA/TNFAIP3 |
| hsa05162 | Measles | 5/28 | 0.00168386134767641 | IL1B/IL6/RELA/TBK1/TNFAIP3 |
| hsa04936 | Alcoholic liver disease | 5/28 | 0.00168386134767641 | ALDH3A2/IL1B/IL6/RELA/TBK1 |
| hsa05169 | Epstein-Barr virus infection | 5/28 | 0.00503593628683049 | CDKN1A/IL6/RELA/TBK1/TNFAIP3 |
| hsa05417 | Lipid and atherosclerosis | 5/28 | 0.00625131977853114 | HSPA5/IL1B/IL6/RELA/TBK1 |
| hsa05166 | Human T-cell leukemia virus 1 infection | 5/28 | 0.00668165128118092 | CDKN1A/CDKN2A/IL6/RELA/ZFP36 |
| hsa05171 | Coronavirus disease - COVID-19 | 5/28 | 0.00717202712626491 | EGFR/IL1B/IL6/RELA/TBK1 |
| hsa05131 | Shigellosis | 5/28 | 0.00832681581929036 | ATG16L1/EGFR/IL1B/RELA/TBK1 |
| hsa05206 | MicroRNAs in cancer | 5/28 | 0.0145056950410827 | CDKN1A/CDKN2A/EGFR/PTGS2/SOCS1 |
| hsa04623 | Cytosolic DNA-sensing pathway | 4/28 | 0.00126117833959565 | IL1B/IL6/RELA/TBK1 |
| hsa04137 | Mitophagy - animal | 4/28 | 0.00168386134767641 | GABARAPL2/RELA/TBK1/ULK1 |
| hsa05212 | Pancreatic cancer | 4/28 | 0.001756477796643 | CDKN1A/CDKN2A/EGFR/RELA |

**Supplementary Table 5:** The top 5 DEGs of each cluster.

|  | p_val | avg_log2FC | pct.1 | pct.2 | p_val_adj | cluster | gene |
| --- | --- | --- | --- | --- | --- | --- | --- |
| CXCL12 | 0 | 2.73356541 | 0.964 | 0.391 | 0 | 0 | CXCL12 |
| SFRP2 | 0 | 2.45328005 | 0.54 | 0.085 | 0 | 0 | SFRP2 |
| IGF1 | 0 | 2.30677909 | 0.901 | 0.345 | 0 | 0 | IGF1 |
| IGFBP4 | 0 | 1.95205121 | 0.957 | 0.624 | 0 | 0 | IGFBP4 |
| SCG2 | 0 | 1.93427078 | 0.591 | 0.09 | 0 | 0 | SCG2 |
| PRG4 | 0 | 2.67841547 | 1 | 0.961 | 0 | 1 | PRG4 |
| TWISTNB | 0 | 2.03624579 | 0.831 | 0.413 | 0 | 1 | TWISTNB |
| TIMP3 | 0 | 2.02158644 | 0.992 | 0.776 | 0 | 1 | TIMP3 |
| DEFB1 | 0 | 1.98806943 | 0.904 | 0.3 | 0 | 1 | DEFB1 |
| HTRA1 | 0 | 1.73030361 | 0.985 | 0.807 | 0 | 1 | HTRA1 |
| INHBA1 | 0 | 2.67822338 | 0.935 | 0.492 | 0 | 2 | INHBA |
| PRELP | 0 | 1.9490194 | 0.995 | 0.752 | 0 | 2 | PRELP |
| CLU1 | 0 | 1.90522332 | 0.999 | 0.956 | 0 | 2 | CLU |
| TNFAIP61 | 0 | 1.8301725 | 0.988 | 0.743 | 0 | 2 | TNFAIP6 |
| NDUFA4L2 | 0 | 1.75354629 | 0.98 | 0.628 | 0 | 2 | NDUFA4L2 |
| MFAP51 | 0 | 2.44728912 | 0.792 | 0.258 | 0 | 3 | MFAP5 |
| SFRP4 | 0 | 2.3008754 | 0.754 | 0.233 | 0 | 3 | SFRP4 |
| IGFBP6 | 0 | 1.87818257 | 0.987 | 0.791 | 0 | 3 | IGFBP6 |
| FAM180B1 | 0 | 1.01902738 | 0.619 | 0.166 | 0 | 3 | FAM180B |
| DCN1 | 3.12E-290 | 1.11455628 | 1 | 0.928 | 6.68E-286 | 3 | DCN |
| MGP2 | 4.58E-174 | 1.18886814 | 1 | 0.947 | 9.81E-170 | 4 | MGP |
| CRTAC12 | 7.39E-149 | 0.93338725 | 0.998 | 0.886 | 1.58E-144 | 4 | CRTAC1 |
| OGN1 | 1.73E-139 | 1.1159612 | 0.868 | 0.544 | 3.70E-135 | 4 | OGN |
| CDO12 | 4.09E-134 | 1.09877633 | 0.902 | 0.642 | 8.77E-130 | 4 | CDO1 |
| LUM2 | 1.19E-101 | 0.77873229 | 1 | 0.939 | 2.55E-97 | 4 | LUM |
| RNASE1 | 0 | 4.58137435 | 0.984 | 0.2 | 0 | 5 | RNASE1 |
| C1QA | 0 | 4.5547054 | 0.99 | 0.149 | 0 | 5 | C1QA |
| C1QB | 0 | 4.26260907 | 0.977 | 0.127 | 0 | 5 | C1QB |
| C1QC | 0 | 4.11407316 | 0.974 | 0.085 | 0 | 5 | C1QC |
| MARCO | 0 | 3.77816552 | 0.915 | 0.083 | 0 | 5 | MARCO |
| COL1A12 | 0 | 2.58991179 | 0.985 | 0.681 | 0 | 6 | COL1A1 |
| COL3A11 | 0 | 2.55265912 | 0.984 | 0.746 | 0 | 6 | COL3A1 |
| DGKI | 0 | 0.70191741 | 0.379 | 0.041 | 0 | 6 | DGKI |
| SPARC1 | 1.66E-304 | 1.91088122 | 0.996 | 0.831 | 3.56E-300 | 6 | SPARC |
| COL6A12 | 1.66E-273 | 1.42278914 | 0.999 | 0.813 | 3.56E-269 | 6 | COL6A1 |
| RSPO3 | 7.38E-133 | 0.47721552 | 0.281 | 0.031 | 1.58E-128 | 7 | RSPO3 |
| EFEMP12 | 9.64E-129 | 1.49911097 | 0.997 | 0.646 | 2.07E-124 | 7 | EFEMP1 |
| STEAP41 | 8.12E-115 | 1.28077975 | 0.751 | 0.266 | 1.74E-110 | 7 | STEAP4 |
| APOE1 | 4.35E-106 | 1.44760036 | 0.523 | 0.132 | 9.31E-102 | 7 | APOE |
| CXCL121 | 1.25E-96 | 1.24643069 | 0.923 | 0.466 | 2.69E-92 | 7 | CXCL12 |
| HLA-DPA11 | 0 | 4.18782554 | 1 | 0.288 | 0 | 8 | HLA-DPA1 |
| FCER1A | 0 | 3.97463904 | 0.852 | 0.03 | 0 | 8 | FCER1A |
| HLA-DQA11 | 0 | 3.95352251 | 0.978 | 0.096 | 0 | 8 | HLA-DQA1 |
| HLA-DQB11 | 0 | 3.95183451 | 0.987 | 0.133 | 0 | 8 | HLA-DQB1 |
| HLA-DRB11 | 0 | 3.82993045 | 1 | 0.293 | 0 | 8 | HLA-DRB1 |
| SELE | 0 | 3.6022645 | 0.501 | 0.023 | 0 | 9 | SELE |
| DARC | 0 | 3.34978501 | 0.71 | 0.019 | 0 | 9 | DARC |
| PLVAP | 0 | 3.33348426 | 0.872 | 0.024 | 0 | 9 | PLVAP |
| EMCN | 0 | 2.56958002 | 0.93 | 0.01 | 0 | 9 | EMCN |
| CALCRL | 0 | 2.56952073 | 0.925 | 0.052 | 0 | 9 | CALCRL |
| RGS5 | 0 | 4.98365955 | 0.95 | 0.053 | 0 | 10 | RGS5 |
| ACTA2 | 0 | 4.54085042 | 0.939 | 0.169 | 0 | 10 | ACTA2 |
| TINAGL11 | 0 | 2.2733702 | 0.816 | 0.03 | 0 | 10 | TINAGL1 |
| MYH11 | 0 | 2.24820868 | 0.482 | 0.019 | 0 | 10 | MYH11 |
| PPP1R14A | 0 | 2.2000837 | 0.719 | 0.008 | 0 | 10 | PPP1R14A |
| CD521 | 0 | 3.23088596 | 0.793 | 0.04 | 0 | 11 | CD52 |
| CCL5 | 0 | 3.04205234 | 0.531 | 0.02 | 0 | 11 | CCL5 |
| IL7R1 | 0 | 2.61640483 | 0.698 | 0.023 | 0 | 11 | IL7R |
| KLRB1 | 0 | 2.50038229 | 0.341 | 0.003 | 0 | 11 | KLRB1 |
| CD2 | 0 | 2.38180872 | 0.648 | 0.007 | 0 | 11 | CD2 |
| CPA3 | 0 | 3.57948215 | 0.968 | 0.01 | 0 | 12 | CPA3 |
| CD692 | 0 | 3.52078844 | 0.824 | 0.031 | 0 | 12 | CD69 |
| RGS13 | 0 | 2.71824 | 0.824 | 0.014 | 0 | 12 | RGS13 |
| CTSG | 0 | 2.17541034 | 0.512 | 0.01 | 0 | 12 | CTSG |
| SLC18A2 | 0 | 2.07506789 | 0.76 | 0.003 | 0 | 12 | SLC18A2 |
| CENPF | 0 | 2.55991529 | 0.904 | 0.024 | 0 | 13 | CENPF |
| TOP2A | 0 | 2.14761684 | 0.981 | 0.01 | 0 | 13 | TOP2A |
| KIAA0101 | 0 | 2.05612489 | 0.846 | 0.02 | 0 | 13 | KIAA0101 |
| UBE2C | 0 | 2.01938768 | 0.846 | 0.008 | 0 | 13 | UBE2C |
| TK1 | 0 | 2.01478545 | 0.865 | 0.012 | 0 | 13 | TK1 |
